# GLAG theory for superconducting property variations with A15 composition in Nb_3_Sn wires

**DOI:** 10.1038/s41598-017-01292-4

**Published:** 2017-04-25

**Authors:** Yingxu Li, Yuanwen Gao

**Affiliations:** 1 Key Laboratory of Mechanics on Environment and Disaster in Western China, Lanzhou University, The Ministry of Education of China, Lanzhou, Gansu 730000 P.R. China; 2 Department of Mechanics and Engineering Science, College of Civil Engineering and Mechanics, Lanzhou University, Lanzhou, Gansu 730000 P.R. China; 3 Department of Engineering Mechanics, School of Mechanics and Engineering, Southwest Jiaotong University, Chengdu, Sichuan 610031 P.R. China

## Abstract

We present a model for the variation of the upper critical field *H*_c2_ with Sn content in A15-type Nb-Sn wires, within the Ginzburg-Landau-Abrikosov-Gor’kov (GLAG) theory frame. *H*_c2_ at the vicinity of the critical temperature *T*_c_ is related quantitatively to the electrical resistivity *ρ*, specific heat capacity coefficient *γ* and *T*_c_. *H*_c2_ versus tin content is theoretically formulated within the GLAG theory, and generally reproduces the experiment results. As Sn content gradually approaches the stoichiometry, A15-type Nb-Sn undergoes a transition from the dirty limit to clean limit, split by the phase transformation boundary. The *H*-*T* phase boundary and pinning force show different behaviors in the cubic and tetragonal phase. We dipict the dependence of the composition gradient on the superconducting properties variation in the A15 layer, as well as the curved tail at vicinity of *H*_c2_ in the Kramer plot of the Nb_3_Sn wire. This helps understanding of the inhomogeneous-composition inducing discrepancy between the results by the state-of-art scaling laws and experiments.

## Introduction

At present, conventional low-temperature superconductors such as Nb_3_Sn have been extensively applied in high-energy and nuclear physics, as well as in magnetic resonance imaging systems^1^. Inhomogeneity of Sn content is inevitable in practical Nb_3_Sn conductors, due to the high vapor pressure of Sn at the formation temperature of the A15 phase in a solid-state diffusion reaction^2, 3^. The tin variation in a conductor covers nearly the entire A15 phase field of binary Nb_1−*β*_Sn_*β*_ with *β* = 0.18~0.255^4^. In a Nb_3_Sn conductor, the Sn gradient across the A15 layer has a remarkable impact on the local variation of the superconducting properties^5^. Experiments show that, the upper critical field *B*_c2_ varies almost linearly at ~5 T per at% between 19.5% and ~24 at% Sn, and the transition temperature *T*_c_ versus tin concentration *β* also exhibits a linear relation within nearly the entire A15 phase field^3^. When *β* approaching stoichiometry of Nb_1−*β*_Sn_*β*_, *β* ≈ 24 at%, *T*_c_ (or *B*_c2_) versus *β* no longer keeps the linear relationship due to the lattice softening (decreasing in phonon frequency)^6^, which means the varying Sn content leads to the cubic-tetragonal phase transformation of A15 lattices. The alloying addition (Ti and/or Ta), which is introduced in most modern high-field Nb_3_Sn conductors for increasing the electrical resistivity, suppressing the martensitic phase transformation and thus raising *B*_c2_, also becomes approximately linear with the Sn content *β* ^7^. As for other additives, the ZrO_2_ precipitates in Nb_3_Sn wires could refine Nb_3_Sn grain size such that change the pinning behavior^8^.

For Nb_3_Sn wires, the scaling law and the experiment show a disagreement in the field dependence of the pinning force at high reduced fields^9^. One of the reasons could be the inhomogeneity of microstructure and composition^9^. This also explains the observation that the scaling field lies below the experimental *B*_c2_ of Nb_3_Sn wires. In fact, the scaling field for the critical current reflects the average properties over the wire volume; it thus relates to the local variation of the critical field dependent on the composition gradient^10^. Cooley and the coauthors simulate the effect of Sn composition gradients on the superconducting properties of powder-in-tube (PIT) Nb_3_Sn strand, with an ideal structure modeled by concentric shells with varying Sn content^7^. They found that different Sn profiles have a pronounced effect on the irreversibility fields defined by the extrapolation of Kramer plots *H*_K_, and also that Sn gradients reduce the elementary pinning force, *H*_K_ and the critical current density *J*_c_^7^.

The temperature dependence of the upper critical field *H*_c2_(*T*) in inhomogeneous Nb_3_Sn conductors, as the field-temperature phase boundary, is comprehensively investigated by Godeke *et al*.^2^. It is concluded that, the complete field-temperature phase boundary can be described with the simplest form of the Maki-DeGennes (MDG) relation, and independent of compositional variation, measuring technique, criterion for the critical field and strain state^2^.

Various experiments^11–13^ and models^14, 15^ have recently been conducted to investigate the dependence of the superconductivity and magnetic properties of Nb_3_ Sn samples on Sn content and disorder. The underlying physics for the superconducting properties variation with the A15 composition in Nb_3_Sn is however still not very clear. The already-existing physical formulas for this dependence are insufficient for describing the state-of-art experimental results of binary Nb_3_Sn samples and practical Nb_3_Sn wires. In this paper, we develop a series of formulas based on GLAG theory; with respect to the previous formulas, the present ones systematically describe the major behaviors of the relevant experimental phenomena: the composition dependence of *H*_c2_, phase boundary and pinning behaviors. By this way, new physical insights into the importance of the composition inhomogeneity are provided. As for practical engineering significance, tin composition and possible additives are important for the very high *J*_c_ now achieved in commercial strands^16, 17^. Therefore, describing the superconducting properties dependence on the A15 composition in theory will facilitate the understanding of the optimization for the critical current density *J*_c_. Based on the above considerations, it is of importance to investigate the physical mechanism of the superconducting properties dependences in inhomogeneous Nb_3_Sn samples.

In this paper, We model the variation of the upper critical field *H*_c2_ with Sn content in A15-type Nb_1−*β*_Sn_*β*_, within the GLAG theory frame. In this theory, the occurrence of *H*_c2_ is due to the breaking of orbital pair and the Pauli paramagnetic limiting. Two scattering mechanisms should be considered in type-II superconductors like Nb_3_Sn: electron-transport scattering by impurity (disorder) and spin-orbit scattering. As for A15 Nb_3_Sn, composition inhomogeneity and its deviation from stoichiometry may cause defect and site disorder in Nb_3_Sn lattices^4^, which contribute most to the scattering by impurity. Based on this physical picture, *H*_c2_ is correlated to the superconductivity parameters (the coherence length, the London penetration depth and etc.) as well as the scattering characteristics (the mean free path of electron transporting). As for the strong-coupling superconductor like Nb_3_Sn, one should include the correction for electron-phonon interaction to the weak-coupling BCS value. Since the microscopic parameters mentioned above cannot be determined directly, we correlate them to the transition temperature, normal-state resistivity and coefficient of electronic heat capacity. The three material parameters have been extensively measured as a function of tin content^3, 4, 18, 19^. In this manner, we can determine the superconducting properties variation with composition concentration. The following section will present the detail.

## Model

### Upper critical field at vicinity of superconducting transition temperature

The best quality Nb_3_Sn samples, with highest transition temperature *T*_c_’s and resistance ratios (*RRR* = *ρ*(300 K)/*ρ*(20 K)), have very narrow resistive transitions^19^. The transitions tend to broaden in high fields. Measured ternary PIT wire also exhibits a narrow transition at a wide range of fields and temperatures^2^. At the vicinity of *T*_c_, the upper critical fields *H*_c2_(*T*) are thus nearly the same, and almost independent on the selected critical-state criterion. We are then allowed to determine *H*_c2_ near *T*_c_ uniquely. The resistivities *ρ* near *T*_c_ are approximated as *ρ*(*T*_c_), measured at temperatures just above *T*_c_, and the coefficient of electronic heat capacity *γ* remains a constant at the vicinity of *T*_c_, satisfying the low-temperature heat capacity formula without undergoing a specific heat jump.

In the microscopic physical concept, the scattering of impurity (Supplementary Information A and Fig. A1) enters into the superconductivity of type II superconductors by changing the Ginzburg-Landau (GL) parameter *κ* at the vicinity of *T*_c_. We have *κ* in two limiting cases, *κ*_clean_(*T*) = *κ*(*T*_c_)*χ*_1_(*T*) without scattering effect and *κ*_dirty_(*T*) = *κ*_dirty_(*T*_c_)*χ*_2_(*T*) relevant to scattering, where *κ*_clean_(*T*_c_) ≈ 0.96*δ*_L_(0)/*ξ*_0_ and *κ*_dirty_(*T*_c_) = 0.72*δ*_L_(0)/*l* (see Supplementary Information B for GLAG description of the superconductivity parameters). Here, *δ*_L_(0) is the London penetration depth of the magnetic field at 0 K. *ξ*_0_ is the standard coherence length. *l* is the mean free path of electron transporting. *κ*_clean_ and *κ*_dirty_ refer to the clean limit (*ξ*_0_ ≪ *l*) and dirty limit (*ξ*_0_ ≫ *l*), respectively. *χ*_1_(*T*) and *χ*_2_(*T*) represent the temperature dependence of *κ* for the clean limit and dirty limit, respectively; calculations show that they vary little with *T* near *T*_c_, *χ*_1_(*T*) ≈ *χ*_2_(*T*) ≈ 1^20^.

In light of the classic proposal^19, 21^, the superconductivity parameters *δ*_L_(0), *ξ*_0_ and the scattering parameter *l* involved in the GL parameter *κ* can be linked to three independent material parameters, the transport scattering resistivity *ρ*, the low-temperature coefficient of electronic heat capacity *γ* and the superconducting transition temperature *T*_c_. Assuming a spherical Fermi surface and isotropic metal, we are allowed to use the electron conduction formula Eq. (A1) and the electron heat capacity relation Eq. (C1) in the GLAG description of the superconductivity parameters (Supplementary Information C), and then express *l*, *ξ*_0_, *δ*_L_(0), *κ*_clean_ and *κ*_dirty_ as functions of *ρ*, *γ* and *T*_c_, Eqs. (C15)–(A19). Including the correction for the anisotropy in Nb_3_Sn (Supplementary Information D), one must consider the change of Fermi surface shape from the isotropic model, which leads to the corrected expressions, Eqs. (D3)–(D6).

**Figure 1:**
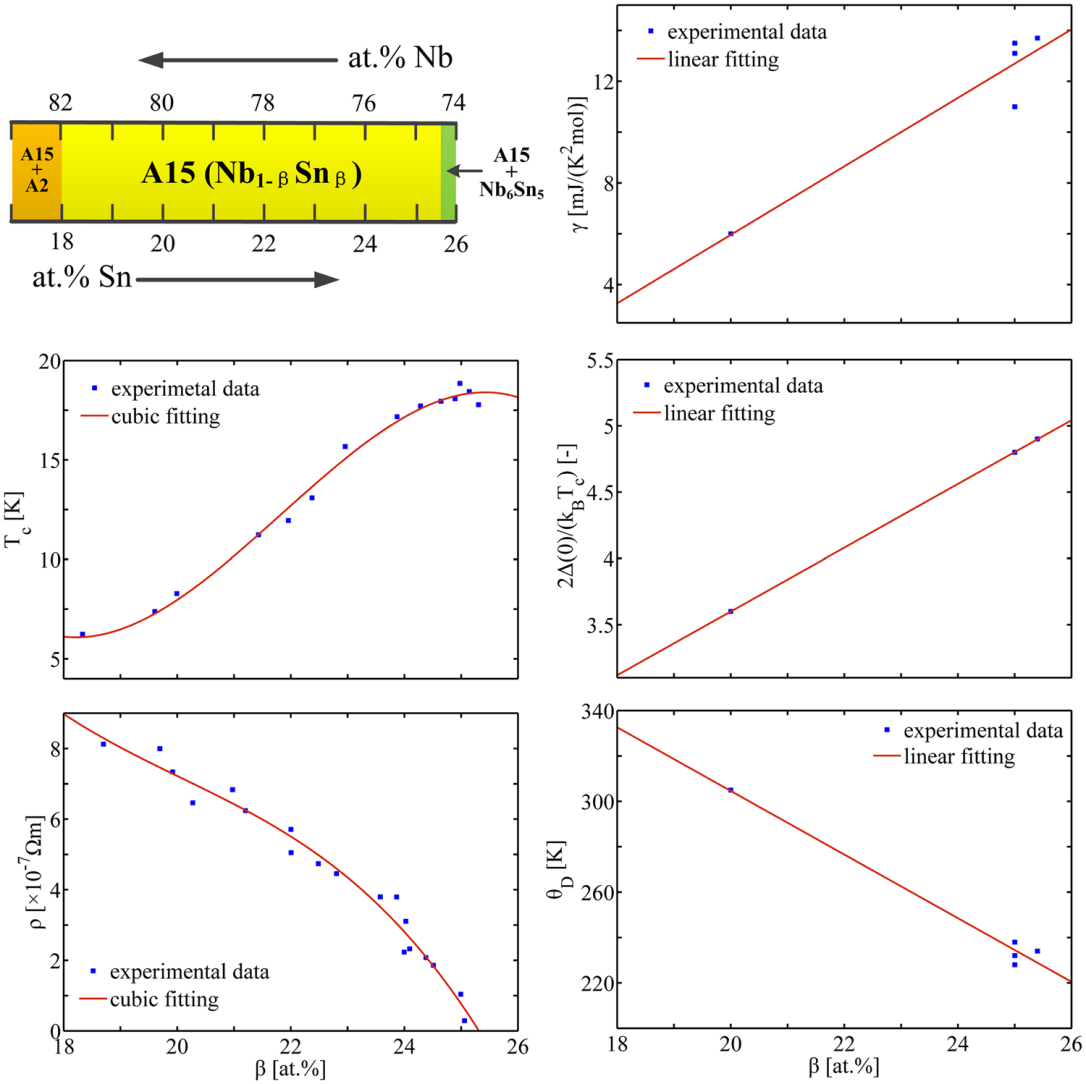
Variations of material parameters *T*_c_, *ρ*, *γ*, *θ*_D_ and 2Δ(0)/(*k*_B_ *T*_c_) with Sn content *β* in the A15 range of binary Nb_1−*β*_Sn_*β*_. The experimental data are extracted from ref. 3. The solid lines represent the global fits to the experimental data in the A15 range.

Let us consider the dependences of *T*_c_, *ρ* and *γ* with Sn content *β*. Experiments^3^ show that *T*_c_ variation with *β* is essentially linear up to ~24 at% ; while between 24 and 25 at% Sn content, *T*_c_ versus *β* has a saturation, Fig. 1. The A15 type lattice undergoes a spontaneous cubic-tetragonal transformation in this range. The lattice softening, as one of the consequences, implies a decreasing in the lattice stiffness (*ħω*_D_ = *k*_B_ *θ*_D_) such that it reduces *T*_c_ according to McMillan *T*_c_ equation^22^ [Eq. (6)]. The normal-state resistivity *ρ*, as measured just above *T*_c_, decreases moderately with increase of *β* within 18~24 at%. Approaching *β* = 25 at%, *ρ*(*β*) exhibits an obviously stronger decrease. Below 24 at%, the coefficient of electronic heat capacity, *γ*, changes linearly with *β* by ~1.5 mJ · K^−2^ · mol^−1^ per at% Sn. Following the treatment to the experimental data in ref. 3, we use global fits to the experimental *T*_c_, *γ* and *ρ* versus *β* in the entire A15 range (Fig. 1 and Table 1),

1 $${T}_{{\rm{c}}},\gamma \,{\rm{or}}\,\rho =\{\begin{matrix}{a}_{1}\beta +{b}_{1}, & {\rm{linear}}\,\mathrm{fit},\\ {a}_{2}{\beta }^{3}+{b}_{2}{\beta }^{2}+{c}_{2}\beta +{d}_{2}, & {\rm{cubic}}\,{\rm{fit}}{\rm{.}}\end{matrix}$$

**Table 1:** Material parameters variation with Sn content *β* [at.%].

*χ*	Linear fitting	(*χ* = *a*_1_ *β* + *b*_1_)		
	*a*_1_	*b*_1_		
*γ*	1.3473	−20.983		
*θ*_D_	−14.018	584.94		
2Δ(0)/(*k*_B_ *T*_c_)	0.24043	−1.2087		
***χ***	**Cubic fitting**	**(** ***χ*** ** = ** ***a***_**2**_ ***β*** ^**3**^ ** + ** ***b***_**2**_ ***β*** ^**2**^ ** + ** ***c***_**2**_ ***β*** ** + ** ***d***_**2**_ **)**		
	***a***_**2**_	***b***_**2**_	***c***_**2**_	***d***_**2**_
*T*_c_	−0.065923	4.3157	−91.612	641.31
*ρ*	−0.021449	1.2923	−26.735	196.6

**Figure 2:**
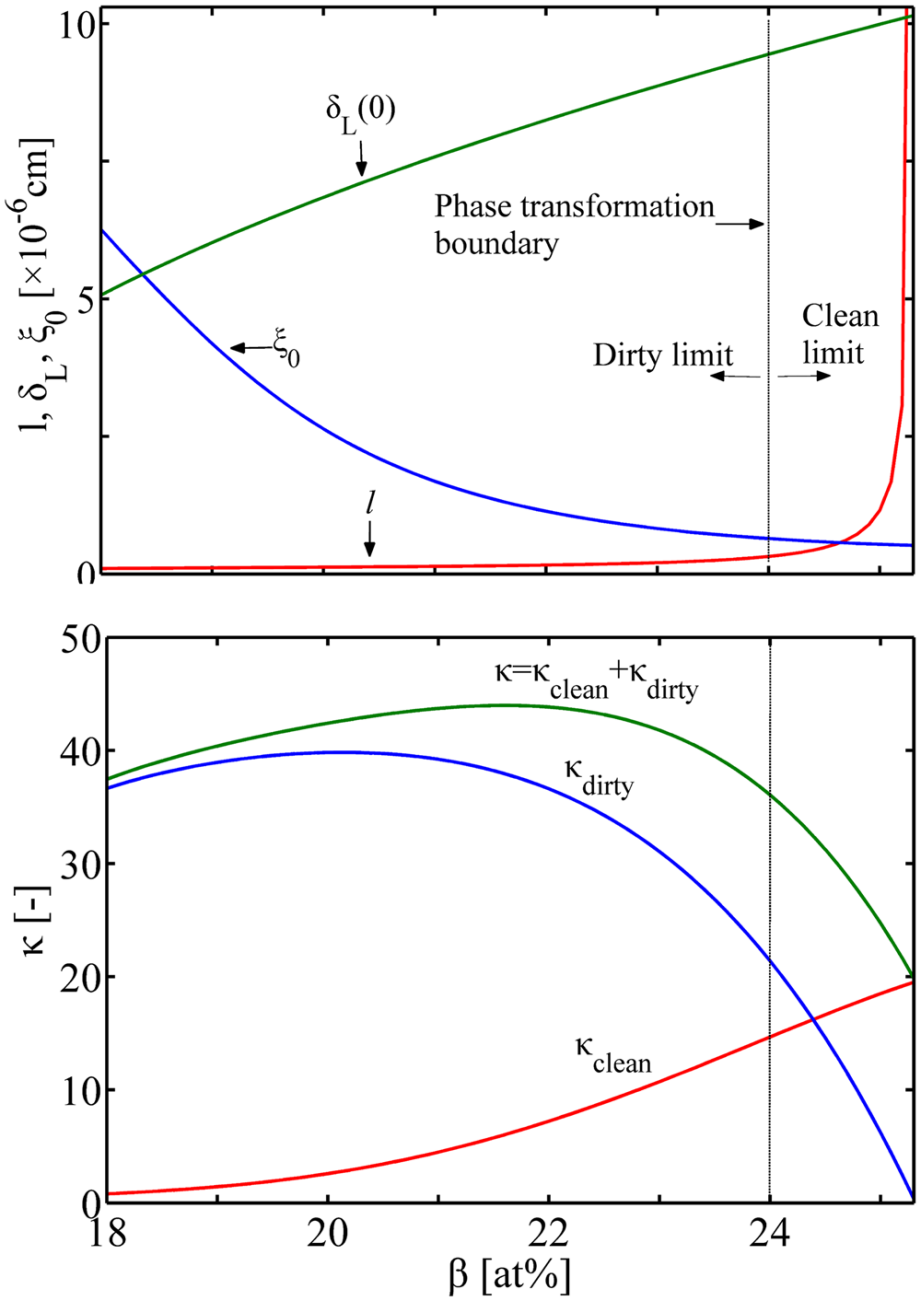
Composition dependence of the London penetration depth *δ*_L_(0), coherence length *ξ*_0_ and electronic mean free path *l* (Top), and the GL parameter *κ* (Bottom).

We are now ready to calculate the dependence of the superconducting characteristic lengths *δ*_L_(0), *ξ*_0_ and *l* with the Sn concentration *β*, by substituting Eq. (1) into Eqs. (D4), (D5) and (D6). The London penetration depth *δ*_L_(0) increases with Sn content, while the coherence length *ξ*_0_ is reduced, Fig. 2. *δ*_L_(0) and *ξ*_0_ are roughly with the same order of magnitude over the A15 range. By comparing the coherence length *ξ*_0_ and the electronic mean free path *l*, one may distinguish the clean limit, the dirty limit and the intermediate state at any A15 composition. At lower Sn content, *ξ*_0_ maintains rather high value compared to *l*; but this difference is mitigated as *ξ*_0_ continues to decrease and *l* increase, for rising *β* until the phase transformation boundary. In the tetragonal phase range, *ξ*_0_ is approximately equal to or even lower than *l*. This implies that, as Sn content gradually approaches the stoichiometry, Nb_1−*β*_Sn_*β*_ undergoes a transition from the “dirty” limit (*ξ*_0_ ≫ *l*) to the “clean” limit (*ξ*_0_ ≪ *l*), and the phase transformation boundary may be taken as the boundary of this transition. In Fig. 2, we also present the variation of GL parameter *κ* with tin content, calculated by Eqs. (C19) and (D3). *κ* in the two limiting cases, *κ*_clean_ and *κ*_dirty_, have the opposite change with Sn content; their change nearly counteract one another in the cubic phase range. While in the tetragonal phase range, *κ*_dirty_ exhibits a more severe decrease compared to the increase in *κ*_clean_. Thus, *κ* = *κ*_clean_ + *κ*_dirty_ varies little in the cubic phase range, but decreases largely in the tetragonal phase.

The magnetic properties of type II superconductors give ${H}_{{\rm{c2}}}=\sqrt{2}\kappa {H}_{{\rm{c}}}$ at the vicinity of *T*_c_, where *H*_c_ is the thermodynamic critical field^23^. Combining this with the expressions *κ* and *H*_c_, Eqs. (C19), (C20) and (D3), renders the upper critical field *H*_c2_ in the clean limit and the dirty limit, Eqs. (C14) and (D7) (see Supplementary Information C and D for detail). Thus, *H*_c2_ at the intermediate *ξ*_0_/*l* can be obtained by summing *H*_c2,clean_ and *H*_c2,dirty_,

2 $${H}_{{\rm{c2}}}={C}_{1}{{T}_{{\rm{c}}}}^{2}(1-T/{T}_{{\rm{c}}}){\gamma }_{{\rm{mJ}}}^{2}+{C}_{2}{T}_{{\rm{c}}}(1-T/{T}_{{\rm{c}}}){\gamma }_{{\rm{mJ}}}{\rho }_{{\rm{\Omega }}{\rm{m}}}.$$

Here, ${C}_{1}=1.979\times {10}^{8}{\pi }^{1/3}c{e}^{-1}{k}_{{\rm{B}}}^{-2}\hslash {({n}^{2/3}S/{S}_{{\rm{F}}})}^{-2}$ and ${C}_{2}=1.356\times {{\rm{10}}}^{-6}{\pi }^{-1}ce{k}_{{\rm{B}}}^{-1}$ in which the physical constants are listed in Table A1. We have omitted multiplier *R*(*λ*_tr_), which is defined in the Gor’kov function^19^ to consider the effect of the *ξ*/*l* variations (ranging from *ξ*/*l* ≪ 1 to *ξ*/*l* ≫ 1), in the sum of *H*_c2,clean_ and *H*_c2,dirty_ [Eq. (2)]. This is reasonable since *R*(*λ*_tr_) changes little within the entire *ξ*/*l* range^24^. The involved three material parameters *T*_c_, *γ*_mJ_ and *ρ*_Ωm_ as well as their variations with the composition concentration in A15 Nb_1−*β*_Sn_*β*_ have been widely measured^3^, Eq. (1) and Fig. 1. Thus, substituting Eq. (1) into Eq. (2), one finds the dependence of the upper critical field *H*_c2_ with Sn content. Note that the above formulas are valid at the vicinity of the superconducting transition temperature *T*_c_.

### Corrections to upper critical field for electron-phonon interaction

A15 type Nb-Sn is a strong-coupling superconductor such that the corrections for electron-phonon (EP) interaction are required. We obtain the upper critical field, Eq. (2), based on the breaking and scattering of Cooper pairs in a weak-coupled interaction. Equation (2) is corrected for EP interaction in two ways: renormalizing the material parameters, and introducing correction parameters to superconductivity itself^19, 22, 25^.

We determine which material parameter should be renormalized in terms of Grimvall’s principle^25^. Both the density of states and the wave function are renormalized by EP interaction, and the electron mass in the absence of EP interactions is replaced by a renormalized electron mass *m* = *m* ^b^(1 + *λ*_ep_), where b refers to band values. The electronic density of states *υ*(*μ*) at Fermi level is renormalized by an enhancement factor 1 + *λ*_ep_ where *λ*_ep_ is the EP interaction parameter, and thus the coefficient of the electronic heat capacity *γ*_mJ_ is enhanced by (1 + *λ*_ep_)*γ*_mJ_. However, *γ*_mJ_ is not changed for EP renormalization in Nb_3_Sn, since there are no EP renormalization effects in the change of the Fermi surface dimensions on alloy and the change in *υ*(*μ*) always depends on the Fermi level and follows almost rigidly any shift in Fermi energy. The electrical resistivity *ρ*_Ωm_ [Eq. (A1)] is not renormalized, since the renormalization of the electron mass *m* exactly cancels against the renormalization of the scattering matrix element as it enters the averaged time *τ* between collisions. We find that, these are implicitly followed by Devantay *et al*.^26^, who do not take renormalizations on *γ*_mJ_ and *ρ*_Ωm_. Thus, we take the only EP correction in Nb_3_Sn by multiplying a factor ${\eta }_{{H}_{{\rm{c2}}}}(T)$ for *H*_c2_,

3 $${H}_{{\rm{c2}}}={\eta }_{{H}_{{\rm{c2}}}}({T}_{{\rm{c}}})[{C}_{1}{T}_{{\rm{c}}}^{2}(1-T/{T}_{{\rm{c}}}){\gamma }_{{\rm{mJ}}}^{2}+{C}_{2}{T}_{{\rm{c}}}(1-T/{T}_{{\rm{c}}}){\gamma }_{{\rm{mJ}}}{\rho }_{{\rm{\Omega }}{\rm{m}}}].$$

Here, ${\eta }_{{H}_{{\rm{c2}}}}({T}_{{\rm{c}}})$ is the ratio of the strong-coupled magnetic pair-breaking parameter to the weak-coupled BCS value, and can be evaluated by the detailed EP spectrum^24^.

If *T*_c_ and the energy gap Δ(0) at 0 K are determined experimentally, we have the strong-coupling correction *η*_Δ(0)_ to Δ(0), *η*_Δ(0)_ = (2Δ(0)/*k*_B_ *T*_c_)_meas_/(2Δ(0)/*k*_B_ *T*_c_)_BCS_ = (2*π*/*η*)^−1^(2Δ(0)/*k*_B_ *T*_c_)_meas_ ≈ 0.283(2Δ(0)/*k*_B_ *T*_c_)_meas_ in which the BCS equation Δ(0) = (*π*/*η*)*k*_B_ *T*_c_ with *η* = 1.78 has been used for the second equal sign. The characteristic (equivalent Einstein) frequency *ω*_0_ [erg] is then determined using^19^ (Supplementary Information E)

4 $${\eta }_{{\rm{\Delta }}(0)}=1+5.3{({\omega }_{0}{k}_{{\rm{B}}}^{-1}{T}_{{\rm{c}}}^{-1})}^{-2}\,\mathrm{ln}({\omega }_{0}{k}_{{\rm{B}}}^{-1}{T}_{{\rm{c}}}^{-1}).$$

The EP correction to *H*_c2_, ${\eta }_{{H}_{{\rm{c2}}}}({T}_{{\rm{c}}})$, can thus be obtained,

5 $${\eta }_{{H}_{{\rm{c2}}}}({T}_{{\rm{c}}})=1+{\pi }^{2}{({\omega }_{0}{k}_{{\rm{B}}}^{-1}{T}_{{\rm{c}}}^{-1})}^{-2}[0.6\,\mathrm{ln}({\omega }_{0}{k}_{{\rm{B}}}^{-1}{T}_{{\rm{c}}}^{-1})-0.26].$$

The another correction parameter (not involved in EP correction for Nb_3_Sn), i.e. the EP interaction parameter *λ*_ep_, is determined by the McMillan strong-coupled *T*_c_ equation^22^

6 $${T}_{{\rm{c}}}=\frac{{\theta }_{{\rm{D}}}}{1.45}\exp (-\frac{1.04(1+{\lambda }_{{\rm{ep}}})}{{\lambda }_{{\rm{ep}}}-{\mu }^{\ast }(1+0.62{\lambda }_{{\rm{ep}}})}),$$

where *θ*_D_ is the Debye temperature and *μ* ^*^ is the pseudo potential parameter for electron Coulomb repulsion (for Nb_3_Sn *μ* ^*^ ≈ 0.2). Note that to obtain a more accurate *λ*_ep_ for Nb_3_Sn one should use the Allen-Dynes *T*_c_ formula, which extends the application range 0 < *λ*_ep_ < 1.5 of Eq. (6) to a large *λ*_ep_ value. Experiments give *λ*_ep_ ≈ 1.8 for Nb_3_Sn. However, the available experimental data is *θ*_D_ and not the characteristic phonon frequency required in the Allen-Dynes *T*_c_ formula. Thus we still apply Eq. (6) at the cost of some accuracy.

The corrections for EP interaction require the information of the Debye temperature *θ*_D_ and the energy gap Δ(0) at 0 K, Eq. (3). Unfortunately, the available data are reported at very limited Sn content. We use linear fits to *θ*_D_ and (2Δ(0)/*k*_B_ *T*_c_) according to the identification^3^ of the gradual changes from strong coupling to weak coupling with decreasing *β*, Fig. 1.

**Figure 3:**
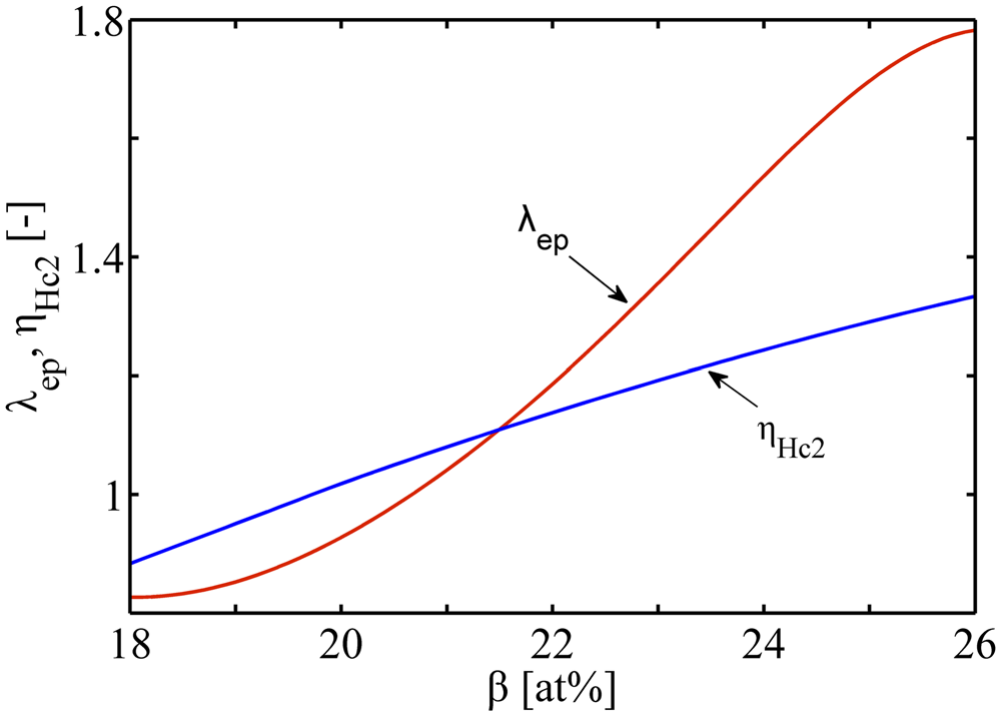
EP correction parameters, the Debye temperature *θ*_D_ and energy gap Δ(0), versus tin content.

Figure 3 presents the calculated EP correction parameters in terms of Eqs. (1), (5) and (6). The result of the strong-coupling correction ${\eta }_{{H}_{{\rm{c2}}}}$ to *H*_*c*2_ approximately equals to the calculated value^19^ of 1.17 at *T*_c_ = 17.8 K. The calculated EP interaction parameter *λ*_ep_ at the stoichiometry 25 at% Sn is slightly lower than the generally accepted value of 1.8^27^. However, at larger derivation from the stoichiometry, the calculated *λ*_ep_ differs relatively larger from the value of 1.8.

### Upper critical field at temperature 0 K

In the following we will concern the behavior of *H*_c2_ at temperatures far from *T*_c_, where the GLAG theory does not apply. This will generalize the above results near *T*_c_ [e.g. Eq. (3)] to a wide temperature range and up to 0 K. The temperature dependence of *H*_c2_ in the scaling law for Nb_3_Sn is determined by an approximate form of the Maki-de Gennes (MDG) relation^28^,

7 $${H}_{{\rm{c2}}}(T)={H}_{{\rm{c2}}}(0)[1-{(T/{T}_{{\rm{c}}})}^{\lambda }].$$

Recent measurements suggest *λ* ≈ 1.52; this value has a universe applicability to a wide range of off-stoichiometric samples and different methods of determining *H*_c2_^10^. This value of *λ* is also the power determined from the MDG theory and Eliashberg theory^28, 29^. Godeke *et al*. demonstrate that the MDG description (7) is universal for *H*_c2_(*T*) relation of Nb_1−*β*_Sn_*β*_ independent of the compositional variation^2, 10^. Recent experiment shows that this relation is applicable for Nb_3_Sn with or without undergoing the cubic-to-tetragonal transition^11^. We are then allowed to extrapolate *H*_c2_ at vicinity of *T*_c_ to *H*_c2_(0) at temperature 0 K for any Sn content. If *H*_c2_(*T*_0_) with *T*_0_ → *T*_c_ has been obtained using Eq. (3), then one can deduce *H*_c2_(0) as

8 $${H}_{{\rm{c2}}}(0)={H}_{{\rm{c2}}}({T}_{0})/[1-{({T}_{0}/{T}_{{\rm{c}}})}^{\lambda }].$$

We assume that EP interaction correction to *H*_c2_ far from *T*_c_ is the same as that for *H*_c2_ near *T*_c_.

WHH (Werthamer-Helfand-Hohenberg) equation, derived from Gor’kov superconductivity theory (Green function method) and taking into account electron spin and spin-orbital scattering^30^, is capable of giving rather satisfactory descriptions for *H*_c2_ behavior of a wide range of commercial and experimental Nb_3_Sn wires. This equation can be written as a simple form

9 $${H}_{{\rm{c2}}}(0)=0.69{H}_{{\rm{c2}}}^{^{\prime} }{T}_{{\rm{c}}},$$

where ${H}_{{\rm{c2}}}^{^{\prime} }=-{(d{H}_{{\rm{c2}}}dT)}_{{T}_{c}}$ ^13^. Substituting the derivative of Eq. (3) into Eq. (9) and then comparing to Eq. (3), one finds *H*_c2_(0) = 0.69*H*_c2_(*T*_0_)/(1 − *T*_0_/*T*_c_). It is further shown that the only difference between WHH equation and MDG relation is the power of (*T*_0_/*T*_c_); Since *T*_0_/*T*_c_ → 1 this difference has very limited impact on the *H*_c2_(0) values. This is consistent with the viewpoint^30, 31^ that MDG relation is a good approximation to Werthamer theory.

**Figure 4:**
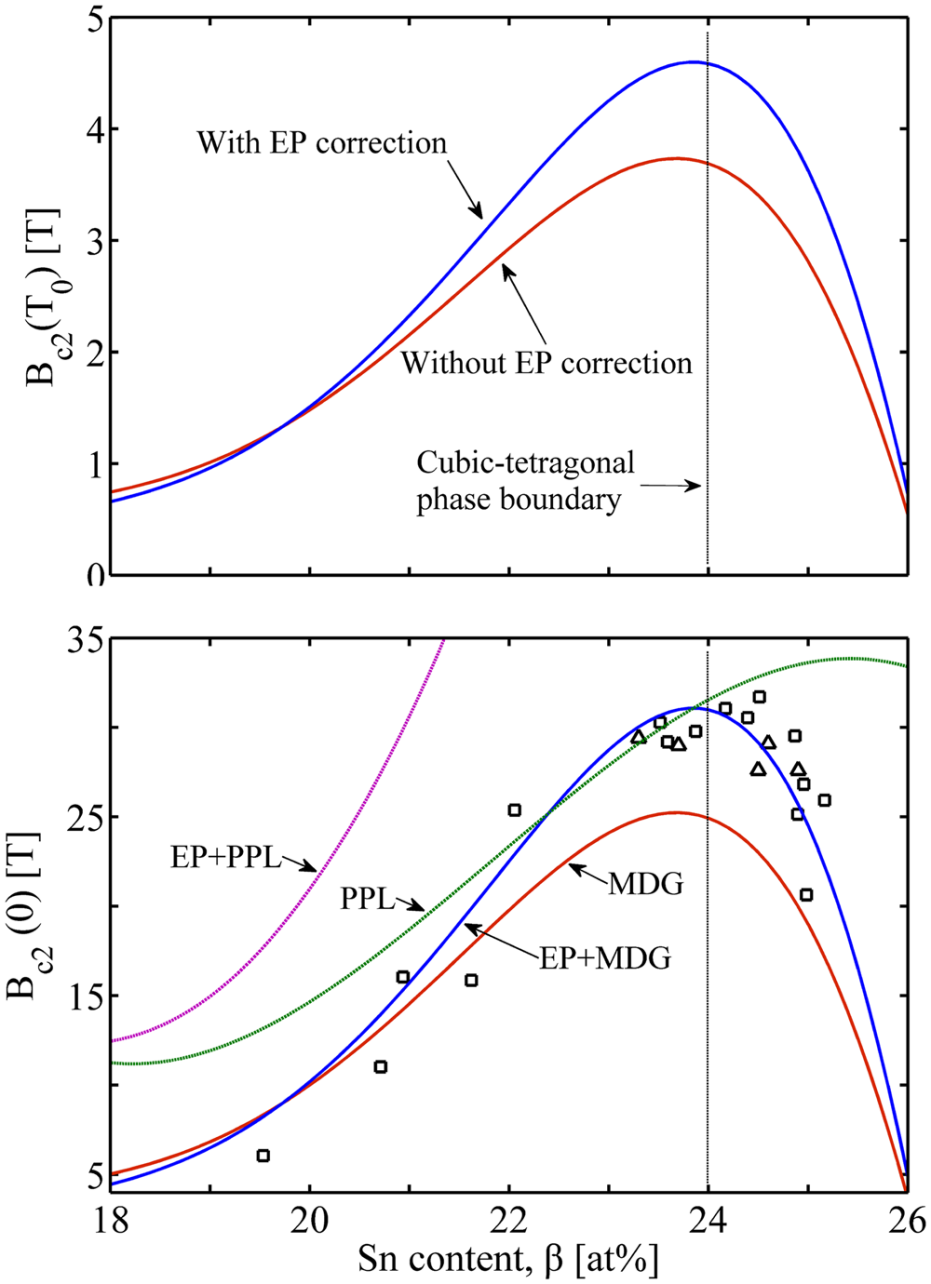
Upper critical field *B*_c2_ variation as a function of tin content *β* at: the vicinity of *T*_c_ (Top) and 0K temperature (Bottom). MDG: extrapolation of *H*_c2_(*T*_c_) with MDG relation, Eqs. (1), (2) and (8); EP + MDG: extrapolation of EP corrected *H*_c2_(*T*_c_) with MDG, Eqs. (1), (2), (3) and (8); PPL: Pauli paramagnetic limit, Eqs. (1) and (10); EP + PPL: EP corrected Pauli paramagnetic limit, Eqs. (1) and (11); □: Experiment dataset-1^3^; △: Experiment dataset-2^11^.

We now calculate the upper critical field *B*_c2_ near *T*_c_ using Eqs. (1), (2) and (3), Fig. 4. The temperature *T*_0_ near *T*_c_ is designated as *T*_0_ = 0.9*T*_c_. One finds the dependence of *B*_c2_(*T*_0_) on Sn content *β* with/without the EP correction. The results of *B*_c2_(*T*_0_) are then substituted into the MDG relation, Eq. (8), to obtain *B*_c2_ at 0 K, *B*_c2_(0). Since the WHH relation is equivalent to the MDG relation, we do not implement it redundantly. The calculation results are impressive (Fig. 4): *B*_c2_(0) obtained by *B*_c2_(*T*_0_) with EP correction and then extrapolated by MDG relation (EP + MDG) is in good agreement with the experiments. This validates the above GLAG descriptions, and also the MDG description for the temperature dependence of the upper critical field at any composition over the A15 phase field.

We find that, the cubic-tetragonal phase boundary at ~24 at% Sn separates the increasing *B*_c2_ versus *β* from the decreasing *B*_c2_ versus *β*. As shown both in the experimental *B*_c2_(0) versus tin content and the EP + MDG curve, the maximum ~29 T of *B*_c2_(0) appears at ~24 at% Sn, and at both sides of the peak, *B*_c2_(0) decreases as tin content deviates more from 24 at% Sn. In the tetragonal phase, *B*_c2_(0) has a more serious reduction; however, at the vicinity of 24 at% Sn, *B*_c2_(0) have nearly the same values at both sides of 24 at% Sn, namely in the cubic phase range and the tetragonal phase range. This is consistent with the experiment by Zhou *et al*.^11^, their tetragonal phase [*B*_c2_(0.3 K) = 29.1 T at *β* = 24.6 ± 0.2 at%] and cubic phase [*B*_c2_(0.3 K) = 29.0 T at *β* = 23.7 ± 0.4 at%] samples exhibiting almost identical *B*_c2_(0) ≈ 29 ± 0.2 T. We infer that, this coincidence occurs in a limited range, where the tin content deviates small from the phase transformation boundary; for a large deviation there is a stronger depression of *B*_c2_(0) in the tetragonal phase. This phenomenon is caused by the underlying relationship between the superconductivity of Nb_3_Sn and the related material parameters; physical properties of the latter is continually changed by the spontaneous cubic-tetragonal transformation.

**Figure 5:**
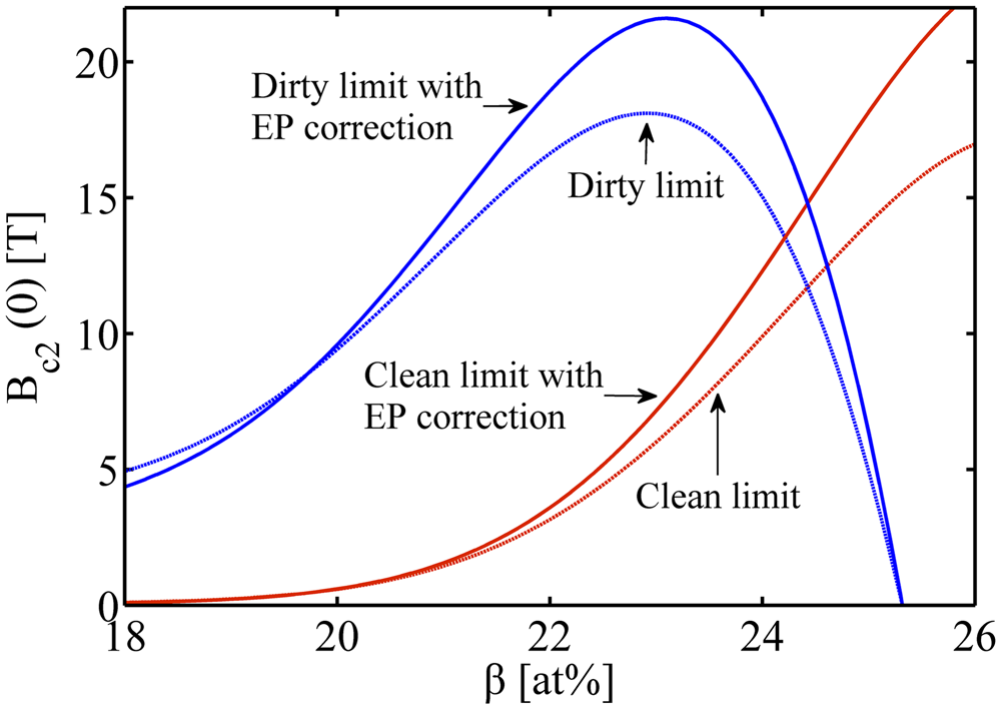
Upper critical field *B*_c2_(0) in the clean and dirty limit case.

We now consider the upper critical field *B*_c2_(0) decomposing into the component in the clean limit case [Eq. (D7)] and that in the dirty limit case [Eq. (C14)], *B*_c2_(0) = *B*_c2,clean_ + *B*_c2,dirty_. One finds that *B*_c2,clean_ increases as *β* raised, but for contrast the dirty one increases up to 24 at% and then deceases drastically, Fig. 5. As we already know, Nb_1−*β*_Sn_*β*_ undergoes a transition from the “dirty” limit to “clean” limit as *β* increased. By comparing Figs 4 and 5, we find that, within 18 at%~24 at%, the component *B*_c2,dirty_ dominates in *B*_c2_(0) and determines the trend of *B*_c2_(0) curve. Once crossing over the phase transformation boundary, *B*_c2,clean_ takes over *B*_c2_(0) variation with *β*.

### Limit for upper critical field: Pauli paramagnetic limit

Preferential Pauli-paramagnetic lowering of normal-state free energy should place a limit on the orbital-pair-breaking *H*_c2_ of filamentary high-field superconductor^32^,

10 $${H}_{{\rm{c2}}}(0)\le {H}_{{\rm{p}}}(0)\equiv 1.84\times {10}^{4}{T}_{{\rm{c}}}[{\rm{Oe}}].$$

This equation is a rough estimation for the limit field *H*_p_(0), since the assumption that the zero temperature difference between superconducting and normal-state magnetizations be at least equal to the Pauli conduction-electron-spin magnetization, is somewhat contrary to experimental results^33^. The correction to *H*_p_(0) for EP interaction is given by

11 $${H}_{{\rm{p}}}(0)=1.84\times {10}^{4}{\eta }_{{H}_{{\rm{c}}}}(0){(1+{\lambda }_{{\rm{ep}}})}^{1/2}{T}_{{\rm{c}}}[{\rm{Oe}}],$$

where ${\eta }_{{H}_{c}}({T}_{c})=1+{\pi }^{2}{({\omega }_{0}{k}_{B}^{-1}{T}_{c}^{-1})}^{-2}[1.1\,\mathrm{ln}({\omega }_{0}{k}_{B}^{-1}{T}_{c}^{-1})+0.14]$ assuming ${\eta }_{{H}_{{\rm{c}}}}(T)$ is independent with temperature^19^.

In Fig. 4, PPL (Pauli paramagnetic limit), Eqs. (1) and (10), slightly lowers the EP + MDG curve after ~23 at% Sn, and it thus provides a good boundary with the experimental data before the phase transformation. This justifies that, PPL is independent with the EP interaction in Nb_3_Sn. In Fig. 4, EP corrected PPL [Eqs. (1) and (11)] is much higher than the experimental curve and has no restriction to *B*_c2_(0). This is consistent with the view by Orlando *et al*.^19^, who demonstrate that EP corrected PPL has nothing to do with Nb_3_Sn superconductivity, since the strong EP interaction in Nb_3_Sn increases largely the Pauli limiting field above its BCS value and the spin-orbit scattering is less involved. This explains why we only take the impurity scattering into account while exclude the spin-orbit scattering.

## Results and Discussion

### Temperature dependence of upper critical field

**Figure 6:**
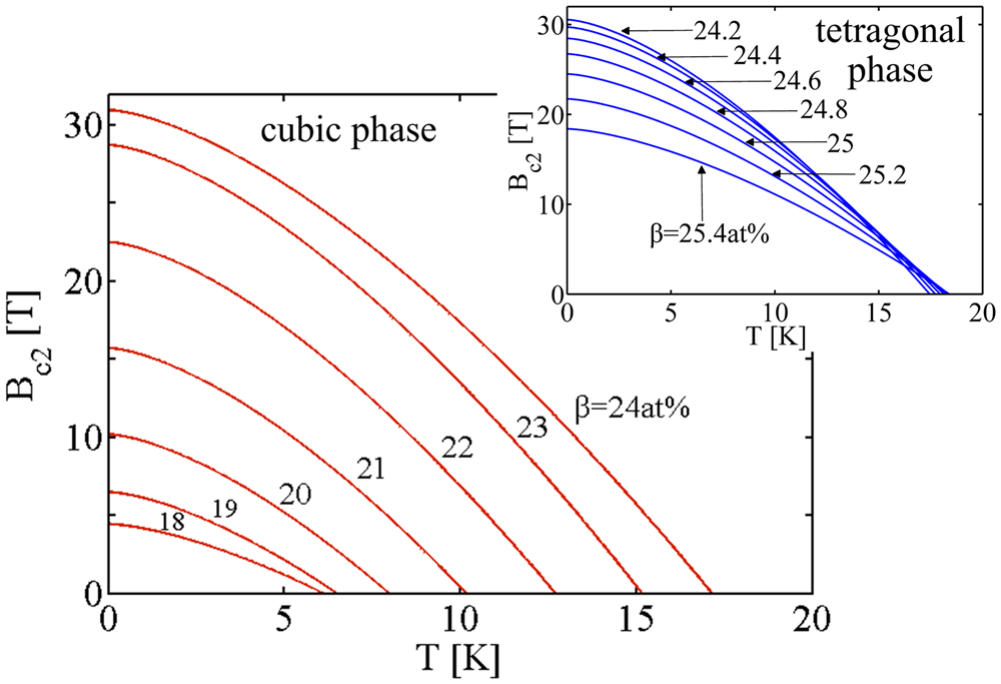
Temperature dependence *B*_c2_(*T*) at varying Sn content in the range of the cubic phase at increment of 1 at% from 18 at% to 24 at% Sn. The insert figure represents the situation in the tetragonal phase for 0.2 at% increment between 24.2 at% and 25.4 at% Sn.

Now, we focus on the temperature dependence of *B*_c2_ at any A15 composition concentration, Eqs. (1), (3), (7) and (8). In Fig. 6, the temperature dependence *B*_c2_(*T*) increases at any temperature as raising the Sn content *β* within the cubic phase range. The tetragonal phase exhibits a reverse behavior, *B*_c2_ at 0 K and at most of other temperatures increasing with reduced Sn content. Orlando *et al*. show that in thin films *B*_c2_(0) is increased with *ρ* rising, however *T*_c_ is suppressed^2, 19^. For *ρ*(*T*_c_) = 35 μΩ · cm, there exists *B*_c2_(0) = 29.5 T and *T*_c_ = 16.0 K; while *ρ*(*T*_c_) = 9 μΩ · cm leads to *B*_c2_(0) = 26.3 T and *T*_c_ = 17.4 K^19^. This corresponds to the calculated composition dependence in the tetragonal phase range, Figs 1 and 6. The measured bulk needle by Godeke *et al*. exhibits a similar behavior: *ρ*(*T*_c_) = 22 μΩ · cm at a 50% normal-state resistance criterion corresponds to*B*_c2_(0) = 27.4 T and *T*_c_ = 16.5 K, and at a 90% criterion *B*_c2_(0) = 28.3 T and *T*_c_ = 16.6 K^2^. A summary of the calculations and the comparisons to experiments is given in Table 2. Through the theoretical expressions, we also figure out the material parameter that is undetermined by experiments, Table 2.

**Table 2:** Calculated *H-T* phase boundary and comparison to experiments.

Samples	*β*[at%] *ρ*(*T*_c_)[μΩ · cm]	*ρ*(*T*_c_)[μΩ · cm]	*γ*[mJ/molK^2^]	*T*_c_[K]	*B*_c2_(0)[T]
Thin films by Orlando *et al*.^19^	−/23.59^(a)^−/24.94	35/359/9.1	−/10.8−/12.6	16.0/16.417.4/18.2	29.5/30.826.3/25.2
Bulk by Godeke *et al*.^2^	−/24.33−/24.33	22(50%)/22.022(90%)/22.0	−/11.8−/11.8	16.5/17.616.6/17.6	27.4/30.128.3/30.1
Polycrystal by Guritanu *et al*.^18^	−/24.46	−/19.4	13.7/11.8	17.8/17.8	25.0/29.4

^(a)^Before the oblique line is the experimental value and after that presents the calculated value.

**Figure 7:**
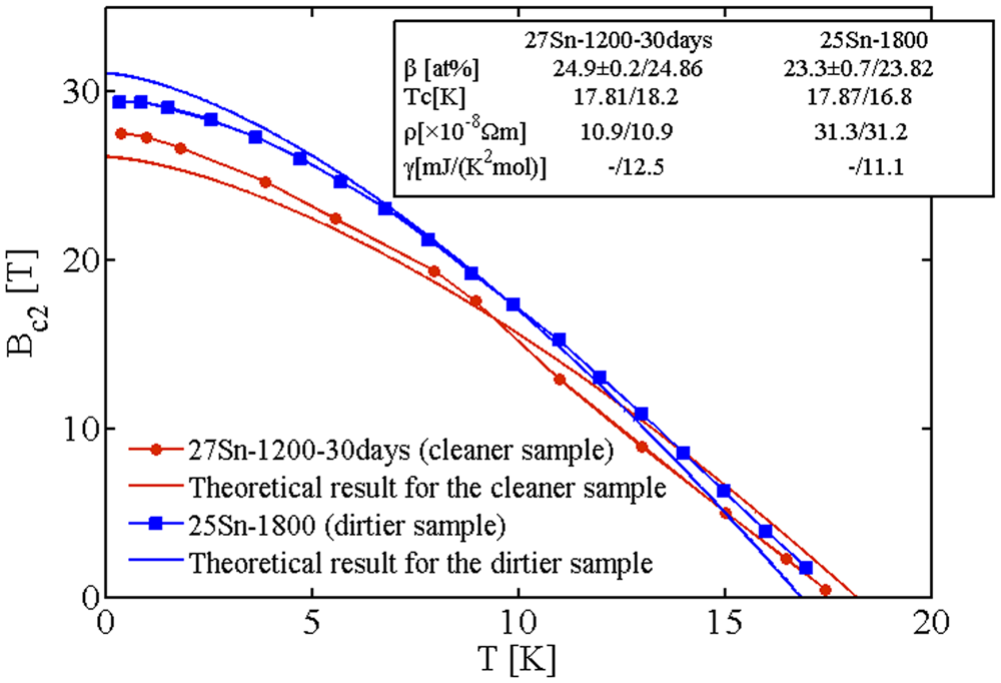
Temperature dependence *B*_c2_(*T*) for two homogenous off-stoichiometric Nb_3_Sn samples, and comparison to experiments^11^.

We also calculate *B*_c2_(*T*) for two rather highly homogenous Nb_3_Sn samples, Fig. 7. Their material parameters, reproduced from^11^, are listed in the insert of the figure. Note that in the absence of the raw experimental value of *γ*, we infer it from Eq. (1). Calculations show that, the dirtier sample (25 Sn-1800) with the larger resistivity (31.3 μΩ · cm) always exhibits a higher *B*_c2_ at any temperature while *T*_c_ is suppressed. This follows the above observation by Orlando *et al*.^19^ and Godeke *et al*.^2^. The little discrepancy from the experiment may be attributed to the absence of the raw data of *γ* and the greater uncertainty in experimentally determining the composition concentration (fluctuation of ±0.7 at% Sn for the dirtier sample compared to ±0.2 at% for the cleaner one).

### Flux pinning force and Kramer plot

We now extend the method for the upper critical field *B*_c2_ to account for the pinning behavior at different composition concentrations. Using the flux pinning model proposed by Kramer^34^, the pinning force per volume for Nb_3_Sn conductors, *F*_p_(*B*), is given by^10^

12 $${F}_{{\rm{p}}}(B)={J}_{{\rm{c}}}B=12.8{\kappa }^{-2}{B}_{{\rm{c2}}}^{2.5}{(B/{B}_{{\rm{c2}}})}^{0.5}{(1-B/{B}_{{\rm{c2}}})}^{2}\,[{\rm{GN}}\cdot {{\rm{m}}}^{-3}],$$

where *J*_c_ is the critical current density. *F*_p_(*B*) is associated with Sn concentration through the composition dependences *B*_c2_(*T*,*β*) and *κ*(*β*), which are formulated by Eqs. (1), (3), (7), (8), (C19) and (D3). The Kramer function ${f}_{{\rm{K}}}(B)={J}_{{\rm{c}}}^{0.5}{B}^{0.25}$ is linear with the magnetic induction *B* and can identify *B*_c2_ at which *f*_K_(*B*) = 0^10^,

13 $${f}_{{\rm{K}}}(B)={J}_{{\rm{c}}}^{0.5}{B}^{0.25}=1.1\times {10}^{5}{\kappa }^{-1}({B}_{{\rm{c2}}}-B)[{{\rm{A}}}^{0.5}{{\rm{m}}}^{-1}{{\rm{T}}}^{0.25}].$$

**Figure 8:**
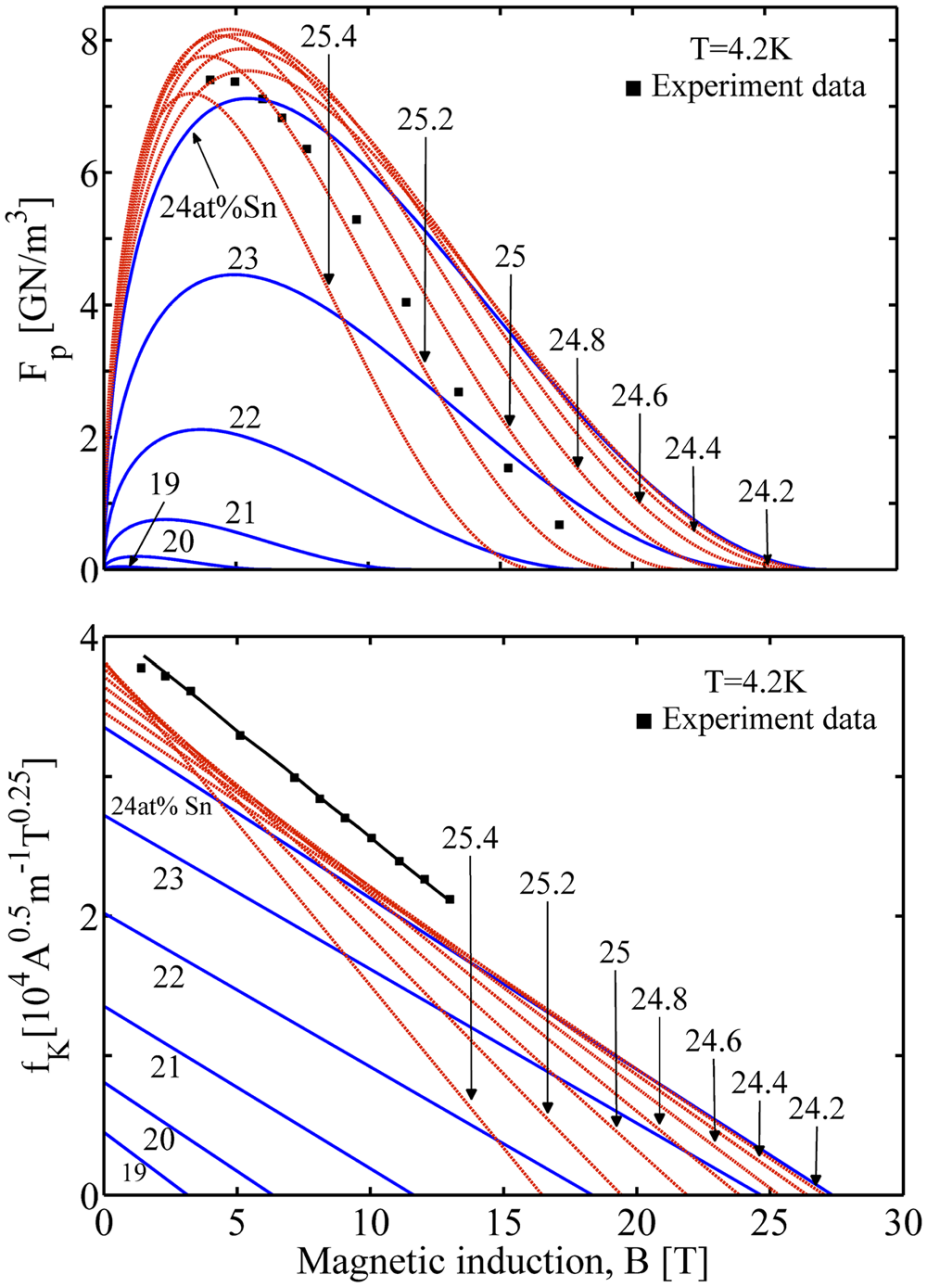
Effect of composition concentration on the magnetic field dependence of the flux pinning force (Top) and Kramer plot (Bottom). The experiment data are extracted from refs 10 and 28.

From Fig. 8 we observe a profound influence of the composition concentration on the field dependent pinning force *F*_p_(*B*). Within the cubic phase range, the increase of Sn content raises the *F*_p_(*B*) remarkably and shifts the peak in each *F*_p_(*B*) curve to the high field region. This is associated with the composition dependent superconducting properties. To be specific, the magnitude of *B*_c2_ determines the position of the peak in *F*_p_(*B*), while the height of *F*_p_(*B*) is related to both *B*_c2_ and *κ*, Eq. (12). *B*_c2_ in the cubic phase range increases as *β* rising (Fig. 4) while *κ* varies little (Fig. 2), thus resulting in the shift of the peak and the increase in the height of *F*_p_(*B*). This case differs from that for the tetragonal phase range, where rising *β* leads to a drastic decrease in both *B*_c2_ and *κ*. As in Fig. 8, the peak shifts to the lower field and the height is nearly the same in all curves (due to ${F}_{{\rm{p}}}(B)\propto {\kappa }^{-2}{B}_{{\rm{c2}}}^{2.5}$). The experimental data of a Nb_3_Sn conductor locate roughly between the calculated *F*_p_(*B*) curves for different Sn contents. This implies that, to describe a real Nb_3_Sn conductor one may consider the composition gradient in the conductor which results in a weighted average of local homogenous composition properties. For Kramer plot, we find its variation consistent with *F*_p_(*B*) for the similar reasons (Fig. 8).

### Composition gradient effect on practical Nb_3_Sn wires

**Figure 9:**
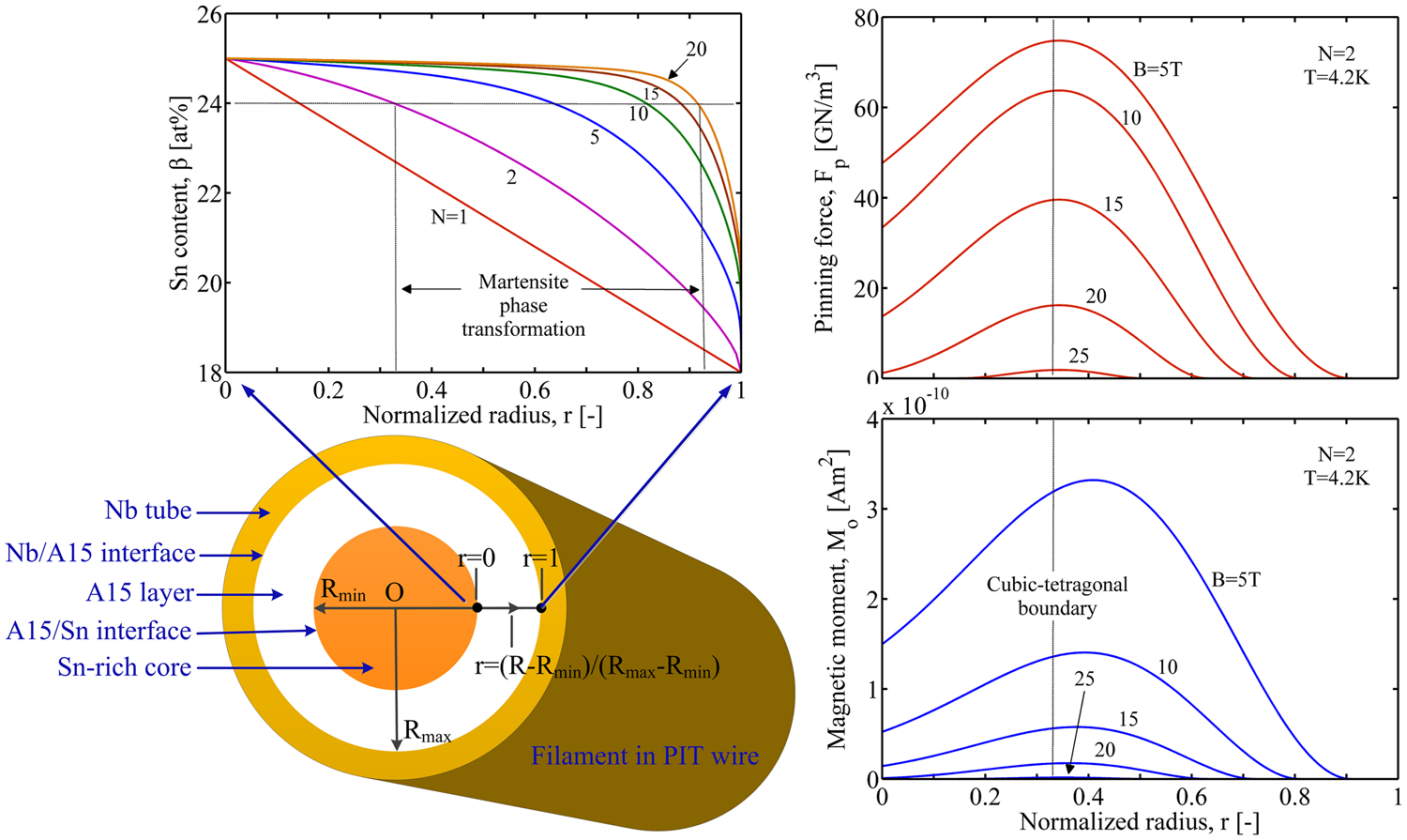
Distribution of the Sn content *β* (Top left), pinning force *F*_p_ (Top right) and magnetic moment *m*_o_ (Bottom right) on the cross section of PIT wire filament (Bottom left), at temperature *T* = 4.2 K. *N* indicates the severity of the overall gradient.

In the following we will apply our descriptions for the superconducting properties variation with tin content in the concentric shells model proposed by Cooley *et al*.^7^, to arrive at the real composition dependent behavior in practical Nb_3_Sn conductors. In the Nb_3_Sn filament of PIT wires, the A15 layer exists between a central Sn-rich core and a coaxial Nb tube, Fig. 9. This structure is convenient for using a series of concentric shells with varying Sn concentration to simulate the composition inhomogeneity in the wire. The Sn content *β* varies with the position in the A15 layer^7^,

14 $$\beta =18+3.5[1-{r}^{N}+{(1-r)}^{1/N}]\,[\mathrm{at} \% ],$$

where *N* indicates the severity and steepness of the overall gradient and *r* is the normalized radius of the filament cross section. The radius *r* is set to be 0 at the A15/Sn-core interface and reaches its maximum 1 at the Nb/A15 interface. The actual position in this area is counted as *R* = 10 + 5*r* [μm], indicating that the inner radius of the A15 layer is *R*_min_ = 10 μm and the outer radius is *R*_max_ = 15 μm.

For PIT wires, the prefactor of the flux pinning force differs from the Kramer model [Eq. (13)], ${F}_{{\rm{p}}}(B)={J}_{{\rm{c}}}B=0.35{B}_{{\rm{c2}}}^{2}{(B/{B}_{{\rm{c2}}})}^{0.5}{(1-B/{B}_{{\rm{c2}}})}^{2}[{\rm{GN}}\cdot {{\rm{m}}}^{-3}]$ and ${f}_{{\rm{K}}}(B)=1.871\times {10}^{4}{B}_{{\rm{c2}}}^{-0.25}({B}_{{\rm{c2}}}-B)[{{\rm{A}}}^{0.5}{{\rm{m}}}^{-1}{{\rm{T}}}^{0.25}]$. We can then link *B*_c2_(*T*,*β*) and *J*_c_ to the radius *r*, and the local magnetic moment is calculated as *m*_o_ = *πR* ^2^ *tLJ*_c_ [Am^2^], where *t* is the thickness of each of the 100 shells, i.e. *t* = (*R*_max_ − *R*_min_)/100 and *L* is the sample length.

Figure 9 presents the Sn content variation with the radius *r* at different gradients *N*. It is found that, larger steepness of the overall gradient *N* has a more drastic decrease near the outer radius (closer to the real situation). The distribution of the magnetic moment *m*_o_ along the radius *r* at different applied magnetic fields is also presented in Fig. 9. At any magnetic field *B*, the regions near the Nb/Nb_3_Sn (*r* = 1) and/or Nb_3_Sn/Sn-core (*r* = 0) interfaces appear no magnetic moment and thus loss of superconductivity. The larger region of the loss occurs for the higher magnetic field, with a suppression in the magnitude of magnetic moment. In fact, the magnetic moment is related to the local critical current density and thus the pinning force *F*_p_(*B*). One can find the Sn content dependence of *F*_p_(*B*) from Fig. 8; in the cubic phase range, *F*_p_(*B*) is raised with the Sn content increasing at any field, and *F*_p_ for lower Sn content more probably turns to disappear at higher fields. These are the underlying reasons for the vanishing of the magnetic moment. The tetragonal phase has a similar corresponding relationship. So, the loss of superconductivity near the boundary of A15 layer is mainly associated with the change of the flux-pinning behavior due to the A15 composition variation.

**Figure 10:**
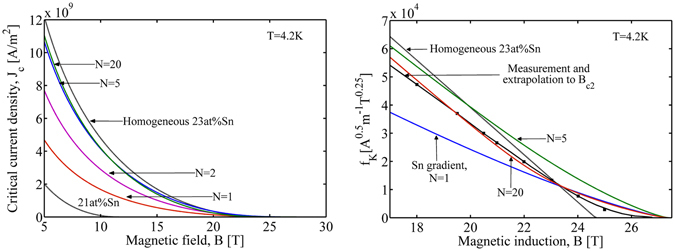
Effect of the composition gradient on the critical current density (Left) and Kramer function ${J}_{{\rm{c}}}^{0.5}{B}^{0.25}$ (Right) of PIT wires. The measurements on SMI ternary PIT wire are duplicated from ref. 2. The Kramer plot with a composition gradient *N* = 20 has a positive curvature approaching the experiment.

Summing the contributions *m*_o_ from each shell results in the total magnetic moment *m*_ot_, which can be measured by magnetometry. The critical current density *J*_c_ of the wire is then expressed as ${J}_{{\rm{c}}}=3{m}_{{\rm{ot}}}/[\pi L({R}_{\max }^{3}-{R}_{\min }^{3})]\,[{A/m}^{{\rm{2}}}]$ in a longitudinal field^7^. In Fig. 10, we present the variation of *J*_c_ as a function of the magnetic field *B* for different Sn gradients *N*. The homogenous sample results are presented to emphasize the effect of the tin gradient. The results of *J*_c_(*B*) allow one to depict the Kramer plot *f*_k_(*B*). The Kramer plot^2^ for the measurements on a SMI ternary PIT wire exhibits an anomalous curvature “tail” at the vicinity of *B*_c2_, Fig. 10. Two calculated homogenous samples with 21 at% and 23 at% appear no curvature, however, the inhomogeneous samples have a curved *f*_k_(*B*) when approaching *B*_c2_. An acceptable agreement with the experiment is found for the conductor with gradient severity *N* = 20. At this point, we reproduce the positive curvature in the Kramer plot of the PIT wire, emphasizing the importance of the composition inhomogeneity in the superconducting properties of practical Nb_3_Sn conductors. The composition dependent pinning force and Kramer function explain the disagreement between the results of the scaling laws and the experiments for practical inhomogeneous conductors.

## Conclusions

Although Nb_3_Sn has been extensively used in fusion engineering area like ITER, the dependence of the superconducting properties with inhomogeneous composition is established incompletely in theory. Based on the GLAG theory frame, we derive a series of expressions for the superconductivity parameters [Eq. (1)] and the upper critical field as a function of the three material parameters [Eq. (2)]. These relations have a complete self-consistent theory basis describing the variation of superconductivity of Nb_3_Sn with Sn content. The corrections for EP interaction are included [Eq. (3)]. The theoretical estimation of *H*_c2_(0) variation with Sn content [Eqs. (3) and (8)], provided with the fits to the material parameters, shows an acceptable agreement with the experiments.

A15-type Nb-Sn undergoes a transition from “dirty” limit (*ξ*_0_ ≫ *l*) to “clean” limit (*ξ*_0_ ≪ *l*) as Sn content gradually approaches the stoichiometry [Eqs. (C19), (D4), (D5), (D6) and (D3)]. The change in superconductivity at the vicinity of the critical temperature and in the related material parameters determines the composition dependence of the upper critical field. The MDG description [Eq. (7)] is universal for describing *H-T* phase boundary over the A15 phase field.

In the cubic phase range, *B*_c2_(*T*) increases with raising Sn content [Eqs. (1), (3), (7) and (8)]. There appears an inverse *B*_c2_(*T*) behavior in the tetragonal phase range. A significant influence of the composition concentration on *F*_p_(*B*) [Eqs. (12) and (13)] curves is observed. Within the cubic phase range, the increase of Sn content raises the *F*_p_(*B*) remarkably and shifts the peak in each *F*_p_(*B*) curve to the right side. The peak shifts to the lower field and the shape is nearly the same in the tetragonal range. This can be explained by the composition dependencies *B*_c2_(*T*,*β*) and *κ*(*β*).

The effect of composition gradient on the superconducting properties of PIT wires is considered by applying the obtained formulas in Cooley’s concentric shells model [Eq. (14)]. The loss of superconductivity near the boundary of A15 layer is mainly associated with the change of the flux-pinning behavior due to the A15 composition variation. The inhomogeneous conductor with gradient severity *N* = 20 predicts well the curved “tail” approaching *B*_c2_ in the Kramer plot. This implies that the composition inhomogeneity is an important factor in the unusual phenomenon of the practical Nb_3_Sn conductors.

However, we cannot yet include the effect of the alloying addition (Ti and/or Ta) and the matrix material (bronze and/or copper) in a ternary Nb_3_Sn wire. The composition gradient is an important factor in the unusual phenomenon of practical Nb_3_Sn conductors but not the unique determinant. The most possible role of the alloying addition taken in Nb_3_Sn is the scattering impurity (Supplementary Information A), which changes the electrical resistivity deeply. We will include this effect in the future work to increase the practical value of the present theory.

## Electronic supplementary material

Supplementary information_LI_GAO_Scientific Reports_GLAG theory for superconducting property variations
