# Supplementary material for: GLAG theory for superconducting property variations with A15 composition in Nb3Sn wires
Source: Sci Rep. 2017 Apr 25;7:1133. doi: 10.1038/s41598-017-01292-4 (PMC5430905; doi:10.1038/s41598-017-01292-4)
Supplement: Supplementary file 1 — Supplementary information_LI_GAO_Scientific Reports_GLAG theory for superconducting property variations [file 41598_2017_1292_MOESM1_ESM.pdf]

Supplementary Information

for "GLAG theory for superconducting property variations with A15 composition in Nb_3Sn wires"

Yingxu Li[1,2,3] and Yuanwen Gao[1,2,a)]

1 Key Laboratory of Mechanics on Environment and Disaster in Western China, The Ministry of Education of China, Lanzhou, Gansu 730000, P. R. China

2 Department of Mechanics and Engineering Science, College of Civil Engineering and Mechanics, Lanzhou University, Lanzhou, Gansu 730000, P. R. China

3 Department of Engineering Mechanics, School of Mechanics and Engineering, Southwest Jiaotong University, Chengdu, Sichuan 610031, PR China

a) Corresponding author: *ywgao@lzu.edu.cn; Tel: 0086 931 891 4359; Fax: 0086 931 891 4561.*

Supplementary Information A: Scattering by impurity in normal metal

Let us first introduce the concept of a mean free path l, which may be expressed as $l = v\tau$ if v signifies the averaged velocity (approximated as Fermi velocity v_F) and τ the averaged time between collisions. For a gas of free electrons, electrical conduction can be regarded as the diffusion of electrons under an external force eE. The mean free path l is a distance travelled by the electrons without undergoing collisions, Fig. A1. The electrical conductivity σ is thus associated with τ in the form of

$$\sigma = e^2 D\upsilon(\mu) = n_e e^2 \tau / m , \tag{A1}$$

where D is the diffusion coefficient $D = lv/3$ and $\upsilon(\mu)$ is the electronic density of states at the Fermi surface [1]. m is the electron mass, e is the unit charge and n_e is the number of electrons per unit volume. The collision depends on certain scattering processes. If the scattering is arisen from impurities and this interaction is elastic and weak, one can obtain the scattering possibility of a system of electrons in the field of impurity centers with Born approximation and the collision integral [2]; consequently, the collision time τ is obtained as $\tau^{-1} = n_i \lambda$ where

$$\lambda = (4\hbar)^{-1}\upsilon(\mu)\int \left| M_{pp'}(\theta) \right|^2 (1-\cos\theta) d\Omega , \tag{A2}$$

and n_i is the number of impurity atoms per unit volume (i.e. impurity concentration). The integration is taken over the surface of Fermi sphere, Ω is the solid angle and θ is the angle between two momentums $\mathbf{p}_0$ ($p_0^{\,2} / 2m = \mu$ where

p_0 is the Fermi momentum and μ is the Fermi energy) and $\mathbf{p}'_0$ on the Fermi surface ($\mathbf{p}$ is fixed and $\mathbf{p}'$ varies with $d\Omega$ during integration). $M_{pp'}(\theta)$ indicates the matrix element of interaction energy of an electron with the impurity.

Supplementary Information B: Coherence length, penetration depth and GL parameter

The coherence length ξ indicates a Cooper pair coherence between electrons extending to a certain distance in a pure superconductor. ξ is determined as $\xi \sim \hbar v_\mathrm{F} / \Delta(T)$ where $\Delta(T)$ is the energy gap in BCS theory [3]. If $T \to T_\mathrm{c}$, then $\Delta \approx 3.06[k_\mathrm{B}^2 T_\mathrm{c}(T_\mathrm{c} - T)]^{1/2}$, where k_B is Boltzmann constant (Table A1) [4]. Use is commonly made of the standard coherence length,

$$\xi_0 = \hbar v_\mathrm{F} / \pi\Delta(0) = (\eta / \pi^2)\hbar v_\mathrm{F} / k_\mathrm{B} T_\mathrm{c} . \tag{B1}$$

The second equal sign holds since the BCS theory gives [4]

$$k_\mathrm{B} T_\mathrm{c} = (2\eta / \pi)\hbar\omega_\mathrm{D} \exp(-2 / g\upsilon) \tag{B2}$$

and

$$\Delta(0) = (\pi / \eta) k_\mathrm{B} T_\mathrm{c} , \tag{B3}$$

where $\eta = 1.78$. We clarify that, the BCS theory holds true for traditional low-T_c superconductors, with the isotropic model of metal and the weak coupling interaction (i.e. $g\upsilon(\mu) << 1$ with the electron-phonon interaction constant $g \sim \hbar^3 / p_0 m$). These conditions are not always fulfilled. For Nb_3Sn with $T_\mathrm{c} = 4.2\mathrm{K}$ and $\hbar\omega_\mathrm{D} = 94.5\mathrm{K}$, one finds $g\upsilon(\mu) = 0.62$ which does not fulfill $g\upsilon(\mu) << 1$. Thus, the BCS theory and its extending results differ from experiment at some extent. However, the disagreement of the theory is remarkably reduced after an appropriate correction [4]. The merit of this theory is the concise physical concepts and brief mathematical representations. The London penetration depth of the magnetic field in a pure superconductor is formulated as $\delta_\mathrm{L} = (mc^2 / 4\pi n_\mathrm{s} e^2)^{1/2}$ according to the definition $\delta_\mathrm{L} = H_0^{-1}\int_0^\infty H dx$. Here, n_s is known as the number of superconducting electrons per volume in contrast to the total number n_e. At the vicinity of T_c, there exists $n_\mathrm{s} / n_\mathrm{e} \approx (T_\mathrm{c} - T) / T_\mathrm{c}$ upon the Ginzburg-Landau equations. From the above discussion, one figures out that both δ_L and ξ have a dependence of $(T_\mathrm{c} - T)^{-1/2}$ at $T \to T_\mathrm{c}$. Hence, the GL parameter κ, defined as the ratio of δ and ξ, tends to a constant at $T \to T_\mathrm{c}$ [5]. For this purpose, we introduce the GL description of κ,

$$\kappa = 2^{3/2} e H_\mathrm{c} \delta^2 / \hbar c , \tag{B4}$$

and the microscopic descriptions of $\delta_\mathrm{L}(T)$ [5],

$$\delta_\mathrm{L}(T) = \delta_\mathrm{L}(0)[1/2(1 - T / T_\mathrm{c})]^{1/2} , \tag{B5}$$

where

$$\delta_\mathrm{L}(0) = (mc^2 / 4\pi n_\mathrm{e} e^2)^{1/2} . \tag{B6}$$

TABLE A1. Dimension, unit and constant value of physical quantities (Gaussian units).[a)]

Physical quantities	Dimension	Unit	Constant value
Boltzmann constant k_B	$L^2MT^{-2}T_E^{-1}$	erg/K	1.381×10^{-16}
Critical temperature T_c	T_E	K	-
Coefficient of electronic heat capacity γ	$L^{-1}MT^{-2}T_E^{-2}$	$erg\cdot cm^{-3}\cdot K^{-2}$	-
Collision time τ	T	s	-
Density of states at Fermi surface $\upsilon(\mu)$	$L^{-5}M^{-1}T^2$	$erg^{-1}\cdot cm^{-3}$	-
Electron mass m	M	g	9.109×10^{-28}
Elementary charge e	$L^{3/2}M^{1/2}T^{-1}$	esu	4.803×10^{-10}
Planck constant $\hbar$	L^2MT^{-1}	$erg\cdot s$	1.055×10^{-27}
Electrons number per unit volume n_e	L^{-3}	cm^{-3}	2.59×10^{23}
Electrical resistivity ρ	T	s	-
Fermi velocity v_F	LT^{-1}	cm/s	-
Flux quantum Φ_0	$L^{3/2}M^{1/2}T^{-1}$	Maxwell	$\pi\hbar c/e$
Light velocity in vacuum c	LT^{-1}	cm/s	2.998×10^{10}
Ratio of Fermi surface S/S_F	Unity	Unity	0.35
Upper critical field H_{c2}	$L^{-1/2}M^{1/2}T^{-1}$	Oe	-
Upper magnetic induction intensity B_{c2}	$L^{-1/2}M^{1/2}T^{-1}$	Gauss	-

[a)] All the formula derivations use the Gaussian units except for the unit conversion for practical application. L, M, T and T_E mean the dimension of length, mass, time and temperature, respectively.

Also the thermodynamic critical field H_c near T_c is formulated as [6]

$$H_c = (16\pi p_0 m/7\zeta_3\hbar^3)^{1/2} k_B T_c (1-T/T_c) , \tag{B7}$$

where the Riemann zeta function $\zeta_3 = \sum_{n=1}^{\infty} n^{-3} \approx 1.2$. Using Eqs. (B5) and (B7) in Eq. (B4) one finds

$$\kappa = (32/7)^{1/2}\pi^{1/2}\zeta_3^{-1/2}\hbar^{-5/2}c^{-1}ek_B m v_F^{1/2}\delta_L^{\,2}(0)T_c \approx 3.459\hbar^{-5/2}c^{-1}ek_B m v_F^{1/2}\delta_L^{\,2}(0)T_c \ . \tag{B8}$$

Substituting ξ_0 for T_c by Eq. (B1), we obtain

$$\kappa = (8/7)^{1/2}\eta\pi^{-2}\zeta_3^{-1/2}\hbar^{-3/2}m^{3/2}v_F^{3/2}n_e^{-1/2}\delta_L(0)/\xi_0 . \tag{B9}$$

Recall that the Fermi momentum p_0 is associated with the number n_e of electrons per volume as [1]

$$p_0 = \hbar(3\pi^2 n_e)^{1/3} , \tag{B10}$$

then we rewrite Eq. (B9) as

$$\kappa = (24/7)^{1/2}\gamma\pi^{-1}\zeta_3^{-1/2}\delta_L(0)/\xi_0 \approx 0.958\delta_L(0)/\xi_0 . \tag{B11}$$

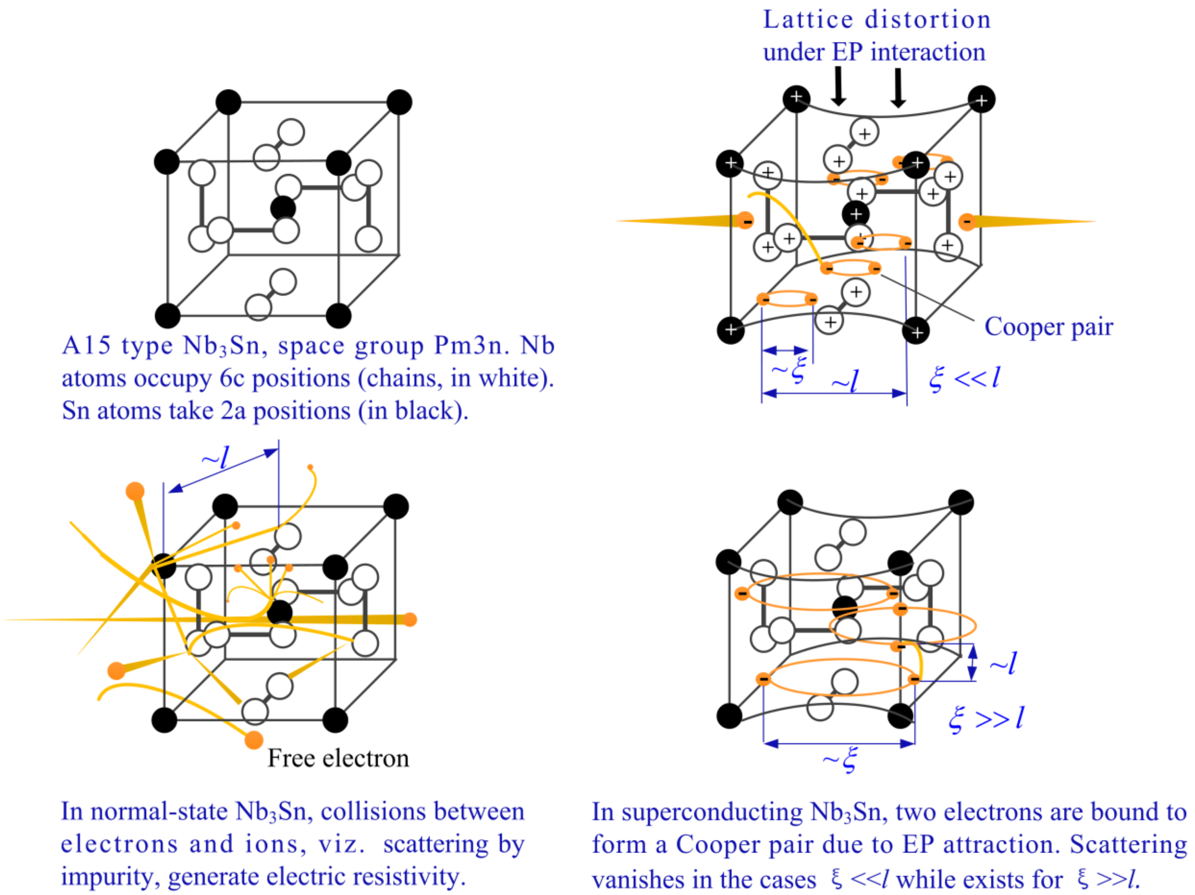

FIG. A1. Schematic of the scattering mechanism in normal-state and superconducting A15-type Nb_3Sn.

Extending to a wide temperature range, κ is formulated as a temperature-dependent relation $\kappa(T) = \kappa(T_c)\chi_1(T)$; calculations show that $\chi_1(T)$ varies little with T [4]. For the case $\xi << l$, scattering from impurity has little impact on the coherence length and the superconductivity [7]. This implies κ remains Eq. (B11) for this case.

It is natural to address the opposite limiting case $\xi >> l$. At first sight, the scattering from impurities reduces the coherence length. Indeed, one could use the diffusion process of an electron in the field of impurity (Supplementary Information A and Fig. A1) to justify the speculation. Recall that under the consideration of scattering, D is the diffusion coefficient with $D = lv/3$ in the diffusion equation $j_e = -D\nabla n_e$. Here, j_e is the diffusion flux, i.e. the number of electrons passing through 1m^2 plane in 1s. It follows from the diffusion equation that $x \sim (Dt)^{1/2}$ where x is the distance travelled by a electron in t. Without scattering and in the same period t, an electron travels a distance of $\xi \sim vt$. Substituting this into x expression, we can obtain the effective coherence length ξ' as $\xi' \sim x \sim (\xi l)^{1/2} \sim (\xi / n_i)^{1/2}$. This is the justification of the above speculation. The scattering of impurity also affects the penetration length δ; the approximation relation is written as $\delta' \sim \delta_L(\xi / l)^{1/2} \sim \delta_L(\xi n_i)^{1/2}$ [4]. Finally, we arrive at the effective GL parameter κ' in the impurity field for $\xi >> l$, $\kappa' = \delta' / \xi' \sim \delta_L / l \sim \delta_L n_i$. Based on a rigorous

gauge-invariant solution of the linearized Gor'kov equations [7], one can obtain a precise relation, $\kappa'(T_c)=0.72\delta_L(0)/l$ and $\kappa'(T)=\kappa'(T_c)\chi_2(T)$. The property of $\chi_2(T)$ is similar with $\chi_1(T)$. We are now ready to summary the dependence of GL parameter κ with temperature and impurity concentration n_i [with the aid of Eqs. (B8) and (B11)],

$$\kappa(T)=\kappa(T_c)\chi_1(T),\ \xi<<l\ \text{(“clean” limit)}, \tag{B12}$$

where $\kappa(T_c)\approx 0.96\delta_L(0)/\xi_0$ or $\kappa(T_c)=3.46\hbar^{-5/2}c^{-1}ek_Bmv_F^{1/2}\delta_L{}^2(0)T_c$.

$$\kappa(T)=\kappa(T_c)\chi_2(T),\ \xi>>l\ \text{(“dirty” limit)}, \tag{B13}$$

where $\kappa(T_c)=0.72\delta_L(0)\lambda n_i v_F^{-1}$ or $\kappa(T_c)=0.72\delta_L(0)/l$. We have omitted the superscript of κ' in Eqs. (B12) and (B13).

The GL theory of magnetic properties of type II superconductors gives that at the vicinity of H_{c2} [8],

$$H_{c2}=\sqrt{2}\kappa H_c. \tag{B14}$$

Supplementary Information C: Relation with material parameters

Now we correlate the electronic density of states, $\upsilon(\mu)$, to the coefficient of electronic heat capacity, γ, upon isotropic model of metal,

$$\gamma=\pi^2k_B{}^2\upsilon(\mu)/3. \tag{C1}$$

The use of isotropic model equation here is reasonable since the experiments do not show evidence of a highly anisotropic Fermi surface for Nb_3Sn [9]. Note that the anisotropy of the physical properties is experimentally identified in compositionally inhomogeneous single-crystal, [10] Fe-based [11] and cuprate [12] superconductors. $\upsilon(\mu)$ may be expressed as a function of the Fermi momentum p_0:

$$\upsilon(\mu)=p_0m/(\pi^2\hbar^3). \tag{C2}$$

Combining Eqs. (C1) and (C2), the Fermi velocity v_F is related to γ in a manner of

$$v_F=3k_B{}^{-2}m^{-2}\hbar^3\gamma. \tag{C3}$$

Let's consider the electrical resistivity ρ, Eq. (A1), and substituting Eqs. (C1) and (C3) into it, one obtains

$$l=3^{-1}\pi^2e^{-2}m^2\hbar^{-3}k_B{}^4\gamma^{-2}\rho^{-1}. \tag{C4}$$

This implies a variation of the electronic average free path l as a function of γ and ρ. We then reformulate the coherence length ξ_0, the number of electrons per unit volume, n_e, and the London penetration depth $\delta_L(0)$ by using Eqs. (B1), (B6) and (B10) in Eq. (C3), respectively,

$$\xi_0=5.34\pi^{-2}m^{-2}k_B{}^{-3}\hbar^4\gamma T_c^{-1}, \tag{C5}$$

$$n_e=9\pi^{-2}m^{-3}k_B{}^{-6}\hbar^6\gamma^3, \tag{C6}$$

$$\delta_L(0)=0.167\pi^{1/2}m^2ce^{-1}k_B{}^3\hbar^{-3}\gamma^{-3/2}. \tag{C7}$$

Now using Eqs. (B12), (B13), (C3), (C4) and (C7), we are allowed to find the dependence of κ on the three independent material parameters γ, ρ and T_c for the dirty limit and clean limit near T_c:

$$\kappa_\mathrm{clean} = 9.43\times10^{-2}\pi^{3/2}m^4ce^{-1}k_\mathrm{B}{}^6\hbar^{-7}\chi_1(T)\gamma^{-5/2}T_\mathrm{c}, \tag{C8}$$

$$\kappa_\mathrm{dirty} = 0.361\pi^{-3/2}cek_\mathrm{B}{}^{-1}\chi_2(T)\gamma^{1/2}\rho. \tag{C9}$$

Further, using Eq. (C3) in Eq. (B7), we express the thermodynamic critical field H_c as

$$H_\mathrm{c} = 2.3905\pi^{1/2}T_\mathrm{c}(1-T/T_\mathrm{c})\gamma^{1/2}. \tag{C10}$$

Substituting Eqs. (C8)-(C10) into Eq. (B14), one finds the upper critical field H_c2 in the two limiting cases,

$$H_\mathrm{c2,clean} = 0.3188\pi^2m^4ce^{-1}k_\mathrm{B}{}^6\hbar^{-7}\chi_1(T)\gamma^{-2}T_\mathrm{c}^2(1-T/T_\mathrm{c})\ [\mathrm{Oe}], \tag{C11}$$

$$H_\mathrm{c2,dirty} = 1.2204\pi^{-1}cek_\mathrm{B}{}^{-1}\chi_2(T)T_\mathrm{c}(1-T/T_\mathrm{c})\gamma\rho\ [\mathrm{Oe}]. \tag{C12}$$

For application convenience we convert ρ in Gaussian unit [s] into $\rho_{\Omega\mathrm{m}}$ $[\Omega\cdot\mathrm{m}]$ in fashion to experiments, using the unit conversion relation $1\mathrm{s}=1\mathrm{esu}\cdot\mathrm{cm}=9.0\times10^{11}\Omega\cdot\mathrm{cm}$ and $\rho_\mathrm{s}=9^{-1}\times10^{-9}\rho_{\Omega\mathrm{m}}$. For γ there is also a conversion relation $\gamma_\mathrm{erg}=1.0\times10^4\gamma_\mathrm{mJ}$ by the relation $1\mathrm{J}\cdot\mathrm{cm}^{-3}\cdot\mathrm{K}^{-2}=1.0\times10^7\mathrm{erg}\cdot\mathrm{cm}^{-3}\cdot\mathrm{K}^{-2}$. As experiments always measure γ in $[\mathrm{mJ}\cdot\mathrm{mol}^{-1}\cdot\mathrm{K}^{-2}]$, γ_mJ $[\mathrm{mJ}\cdot\mathrm{cm}^{-3}\cdot\mathrm{K}^{-2}]$ in the formulas should be further transformed as $\gamma_\mathrm{mJ}\to\gamma_\mathrm{mJ}/V_\mathrm{mol}$ (since $\mathrm{V_{mol}mJ}\cdot\mathrm{cm}^{-3}\cdot\mathrm{K}^{-2}=1\mathrm{mJ}\cdot\mathrm{mol}^{-1}\cdot\mathrm{K}^{-2}$ where V_mol is the volume occupied by 1mol atoms).

For Nb_3Sn, recent specific heat measurements reveal $V_\mathrm{mol}=11.085\pm0.005\ \mathrm{mol}^{-1}\mathrm{cm}^3$ at 10K temperature [13]. Thus, Eqs. (C11) and (C12) are rewritten as

$$H_\mathrm{c2,clean} = C_1\chi_1(T)T_\mathrm{c}^2(1-T/T_\mathrm{c})\gamma_\mathrm{mJ}^{-2}\ [\mathrm{Oe}], \tag{C13}$$

$$H_\mathrm{c2,dirty} = C_2\chi_2(T)T_\mathrm{c}(1-T/T_\mathrm{c})\gamma_\mathrm{mJ}\rho_{\Omega\mathrm{m}}\ [\mathrm{Oe}]. \tag{C14}$$

where $C_1=0.3188\times10^{-8}\pi^2m^4ce^{-1}k_\mathrm{B}{}^6\hbar^{-7}$ and $C_2=1.356\times10^{-6}\pi^{-1}cek_\mathrm{B}{}^{-1}$. We also concern the determination of whether the experimental sample is in dirty limit or not. The same unit conversations are applied to the electronic free path l, the penetration depth $\delta_\mathrm{L}(0)$, the coherence length ξ_0 and the GL parameter κ, and we rewrite Eqs. (C4), (C7), (C5), (C8), (C9) and (C10) into

$$l = 30\pi^2e^{-2}m^2\hbar^{-3}k_\mathrm{B}^4\gamma_\mathrm{mJ}{}^{-2}\rho_{\Omega\mathrm{m}}^{-1}, \tag{C15}$$

$$\xi_0 = 5.34\times10^4\pi^{-2}m^{-2}k_\mathrm{B}{}^{-3}\hbar^4\gamma_\mathrm{mJ}T_\mathrm{c}^{-1}, \tag{C16}$$

$$\delta_\mathrm{L}(0) = 0.167\times10^{-6}\pi^{1/2}m^2ce^{-1}k_\mathrm{B}{}^3\hbar^{-3}\gamma_\mathrm{mJ}{}^{-3/2}, \tag{C17}$$

$$\kappa_\mathrm{clean} = 3.127\times10^{-12}\pi^{5/2}m^4ce^{-1}k_\mathrm{B}{}^6\hbar^{-7}\chi_1(T)\gamma_\mathrm{mJ}{}^{-5/2}T_\mathrm{c}, \tag{C18}$$

$$\kappa_\mathrm{dirty} = 4.011\times10^{-9}\pi^{-3/2}cek_\mathrm{B}{}^{-1}\chi_2(T)\gamma_\mathrm{mJ}{}^{1/2}\rho_{\Omega\mathrm{m}}, \tag{C19}$$

$$H_\mathrm{c} = 2.3905\times10^2\pi^{1/2}T_\mathrm{c}(1-T/T_\mathrm{c})\gamma_\mathrm{mJ}{}^{1/2}. \tag{C20}$$

If experiments give the magnetic flux density B_c2 [T] then it is necessary to implement $B_\mathrm{c2}=(H_\mathrm{c2}[\mathrm{Oe}]\times10^{-4})$ [T] in the above formulas (since $B_\mathrm{c2}\ [\mathrm{T}]=\mu_0\cdot H_\mathrm{c2}[\mathrm{A/m}]$ and $1\mathrm{A/m}=4\pi\times10^{-3}\mathrm{Oe}$ with $\mu_0=4\pi\times10^{-7}\mathrm{T}\cdot\mathrm{m/A}$).

Supplementary Information D: Effective electron mass accounting for anisotropic metal

Accounting for the change of Fermi surface shape due to the transition to the anisotropic model and/or the condensed matter from the isotropic metal model, we employ the formulation of wave vector for the anisotropic metal model:

$$k_{\mathrm{F}} = 3^{1/3}\pi^{2/3}(n_{\mathrm{e}}{}^{2/3}S/S_{\mathrm{F}})^{1/2} \tag{D1}$$

with the de Broglie relation $p_0 = m^* v_{\mathrm{F}} = \hbar k_{\mathrm{F}}$ where m^* is the effective electron mass. Here, S/S_{F} is the ratio of the free Fermi surface to the Fermi surface of a free-electron gas of density n_{e}. Substituting this into Eq. (C3) leads to

$$\begin{aligned} m^* &= 3^{2/3}\pi^{-2/3}k_{\mathrm{B}}{}^{-2}\hbar^2(n^{2/3}S/S_{\mathrm{F}})^{-1/2}\gamma \\ &= 3^{2/3}\times 10^4\pi^{-2/3}k_{\mathrm{B}}{}^{-2}\hbar^2(n^{2/3}S/S_{\mathrm{F}})^{-1/2}\gamma_{\mathrm{mJ}} \end{aligned}. \tag{D2}$$

Substituting this equation into Eq. (C18) one may obtain

$$\kappa_{\mathrm{clean}} = 5.854\times 10^5\pi^{-1/6}ce^{-1}k_{\mathrm{B}}{}^{-2}\hbar(n^{2/3}S/S_{\mathrm{F}})^{-2}\chi_1(T)T_{\mathrm{c}}\gamma_{\mathrm{mJ}}{}^{3/2}. \tag{D3}$$

The following equations can be obtained in the same way:

$$l = 9\times 10^9(3\pi^2)^{1/3}\hbar e^{-2}(n^{2/3}S/S_{\mathrm{F}})^{-1}\rho_{\Omega\mathrm{m}}^{-1}, \tag{D4}$$

$$\xi_0 = 1.234\times 10^{-4}\pi^{-2/3}k_{\mathrm{B}}(n^{2/3}S/S_{\mathrm{F}})\gamma_{\mathrm{mJ}}{}^{-1}T_{\mathrm{c}}^{-1}, \tag{D5}$$

$$\delta_{\mathrm{L}}(0) = 72.257\pi^{-5/6}ce^{-1}\hbar k_{\mathrm{B}}{}^{-1}(n^{2/3}S/S_{\mathrm{F}})^{-1}\gamma_{\mathrm{mJ}}{}^{1/2}, \tag{D6}$$

$$H_{\mathrm{c2,clean}} = 1.979\times 10^8\pi^{1/3}ce^{-1}k_{\mathrm{B}}{}^{-2}\hbar(n^{2/3}S/S_{\mathrm{F}})^{-2}\chi_1(T)\gamma_{\mathrm{mJ}}{}^2T_{\mathrm{c}}{}^2(1-T/T_{\mathrm{c}}). \tag{D7}$$

Supplementary Information E: Lambert *W(X)* function

Equation (4) can be solved via the Lambert $W(X)$ function, which is defined as the solution to $W\exp(W) = X$. $W(K,X)$ is the *K*-th branch of the multi-valued function $W(X)$. It follows that a solution to $y = x^{-2}\ln x$ is $x = \exp[-W(K,-2y)/2]$, where K representing the branch of the multivalued W is selected to ensure positive and real x. For $\omega_0 k_{\mathrm{B}}^{-1}T_{\mathrm{c}}^{-1}$ in question, we arrive at $\omega_0 k_{\mathrm{B}}^{-1}T_{\mathrm{c}}^{-1} = \exp\{-0.5W[K,-0.377(\eta_{\Delta(0)}-1)]\}$ with $K = -1$.

Reference

1 Singleton, J. *Band theory and electronic properties of solids*. (Oxford University Press, 2001).
2 Lifshitz, E. M., Pitaevskii, L. P. & Landau, L. D. *Physical kinetics*. Vol. 60 (Pergamon press, 1981).
3 Bardeen, J., Cooper, L. N. & Schrieffer, J. R. Theory of Superconductivity. *Physical Review* **108**, 1175-1204 (1957).
4 Abrikosov, A. A. *Fundamentals of the Theory of Metals*. (Elsevier, 1988).
5 Gorkov, L. P. Microscopic derivation of the Ginzburg-Landau equations in the theory of superconductivity. *Sov. Phys. JETP* **9**, 1364-1367 (1959).
6 Abrikosov, A. A. Magnetic properties of superconductors of the second group. *Sov. Phys.-JETP (Engl. Transl.);(United States)* **5** (1957).
7 Helfand, E. & Werthamer, N. Temperature and Purity Dependence of the Superconducting Critical Field, Hc2. II. *Physical Review* **147**, 288-294 (1966).
8 Abrikosov, A. Nobel lecture: Type-II superconductors and the vortex lattice. *Reviews of modern physics* **76**, 975-979 (2004).
9 Foner, S. & McNiff Jr, E. Anisotropy of H_{c2} in single crystal Nb_3Sn and V_3Si at high magnetic fields: Limitations of linear chain model. *Physics Letters A* **58**, 318-320 (1976).
10 Roslova, M. *et al.* Crystal growth, transport phenomena and two-gap superconductivity in the mixed alkali metal $(K_{1-z}Na_z)_xFe_{2-y}Se_2$ iron selenide. *CrystEngComm* **16**, 6919 (2014).

11 Hänisch, J. *et al.* High field superconducting properties of Ba(Fe_{1-x}Cox)$_2$$As_2$ thin films. *Scientific Reports* **5**, 17363 (2015).
12 Auslaender, O. M. *et al.* Mechanics of individual isolated vortices in a cuprate superconductor. *Nature Physics* **5**, 35-39 (2009).
13 Guritanu, V. *et al.* Specific heat of Nb_3Sn: The case for a second energy gap. *Physical Review B* **70**, 184526 (2004).
